# High-Efficiency Ultrasound-Guided Regional Nerve Block Workshop for Emergency Medicine Residents

**DOI:** 10.21980/J84P8R

**Published:** 2022-07-15

**Authors:** Brandon Yonel, Eunice Kwak, Mohamad Moussa

**Affiliations:** * University of Toledo College of Medicine and Life Sciences, Department of Emergency Medicine, Toledo, OH

## Abstract

**Audience:**

This small-group workshop is designed for emergency medicine residents. This workshop can also be offered to medical students or faculty interested in reviewing and practicing ultrasound-guided regional nerve blocks.

**Introduction:**

Ultrasound-guided regional nerve block (UGRNB) is a method used to administer local anesthesia to otherwise unreachable locations on the body and may be used in the management of various painful conditions seen in the emergency department. The ultrasound curriculum of our residency program is longitudinal. All residents spend time with the ultrasound director throughout their training, engage in daily bedside ultrasound, and have regular skills training on the various clinical applications of ultrasound. Additionally, all residents have a required ultrasound rotation dedicated to performing bedside ultrasound in the Emergency Department. Although others have outlined approaches to teaching emergency medicine residents the techniques needed to perform UGRNBs, the value of taking a one-day, high-efficiency teaching strategy with a narrow focus on practical application has yet to be appreciated.

**Educational Objectives:**

The objective of this workshop is to provide emergency medicine residents the confidence and skill sets needed to effectively perform five commonly used UGRNBs for conditions encountered in the emergency department. Through this one-day, accelerated workshop, residents will be given an opportunity to sharpen their UGRNB technique prior to applying them in the clinical environment. By the end of this workshop, learners will be able to: 1) recognize the clinical situations in which UGRNBs can be utilized and understand the associated risks, 2) list the commonly used local anesthetic medications and their proper dosing in respect to regional nerve blocks, 3) demonstrate proper ultrasound probe positioning and identify relevant anatomical landmarks for each nerve block on both standardized patients and cadavers, 4) describe the common steps involved to perform each nerve block, 5) perform the five UGRNB techniques outlined in this workshop.

**Educational Methods:**

Small group activity combining didactic learning, case-based learning, and procedural simulation. The didactic component may be delivered in an asynchronous learning or “flipped classroom” format.

**Research Methods:**

In-person interviews of the learners were obtained following the debriefing session during which they were asked about their enjoyment and satisfaction with the workshop. In addition, learners were asked about the value of the activities for their clinical practices and to provide formative feedback regarding the design of the workshop.

**Results:**

Overall, participants reported high levels of enjoyment, and many verbalized their satisfaction with the expeditious and pragmatic nature of the workshop. Some participants commented that they were looking forward to future workshops of similar design. Participants also stated that they felt more confident performing nerve blocks and looked forward to applying these skills in the clinical setting.

**Discussion:**

A focused small-group workshop directed towards developing the confidence and skill set necessary to perform UGRNBs can be successfully offered to emergency medicine residents in a single-day workshop.

**Topics:**

Ultrasound, nerve block, local anesthesia, injection, pain, resident, workshop.

## USER GUIDE

**Table:** 

**List of Resources:**	
Abstract	24
User Guide	26
Small Groups Learning Materials	22
Appendix A: UGRNB Introductory Presentation	35
Appendix B: UGRNB Pearls	36
Appendix C: UGRNB Debrief Instructor Guide	43
Appendix D: UGRNB Post-Workshop Evaluation Survey	44

**Learner Audience:**

Medical Students, Interns, Junior Residents, Senior Residents

**Time Required for Implementation:**

4 hours

**Recommended Number of Learners per Instructor**:

6–12 (depending on resource availability and prior experience of learners)

**Topics:**

Ultrasound, nerve block, local anesthesia, injection, pain, resident, workshop.

**Objectives:**

After completing this small group activity, the learner should be able to:

1. Recognize the clinical situations in which UGRNBs can be utilized and understand the associated risks.
2. List the commonly used local anesthetic medications and their proper dosing in respect to regional nerve blocks.
3. Demonstrate proper ultrasound probe positioning and identify relevant anatomical landmarks for each nerve block on both standardized patients and cadavers.
4. Describe the common steps involved to perform each nerve block.
5. Perform the five UGRNB techniques outlined in this workshop

### Linked objectives and methods

Ultrasound-guided regional nerve blocks (UGRNBs) are a highly efficacious form of pain management that emergency medicine physicians can offer given the proper knowledge and skill set. UGRNBs can be used in the short term to manage the pain of conditions such as rib fractures and shoulder dislocations, in addition to various procedures such as closed reductions. Despite strong evidence in support of efficacy, safety, and ease of use, nerve blocks often remain underutilized in the emergency department setting.^7^ With ultrasound being recognized as a critical tool in the emergency department, the additional skill set needed to perform UGRNBs is marginal and certainly achievable.^8^

Indications for UGRNBs vary widely. Over the past several years, there has been a growing interest in the ultrasound-guided technique and the many advantages that this technique offers for regional anesthesia. Direct visualization of vascular and soft tissue structures with improving UGRNB techniques have contributed to better safety and decreased risk of injuries to important structures during the procedure.

Although they are considered to have a low risk profile of adverse effects, it is still essential to be familiar with potential complications that may arise from performing UGRNBs. Peripheral nerve injury is a rare but possible complication of peripheral nerve blocks. Fortunately, most injuries are transient and oftentimes present as mild mononeuropathies. Additionally, with the use of ultrasound guidance, studies have shown that intraneural injections do not necessarily result in permanent injury. Similarly, vascular puncture leading to blood vessel injury is another infrequent complication of peripheral nerve blocks. One study reported a vascular puncture incidence of 5.7% and 6.6% for femoral and sciatic catheters, respectively. However, studies suggest that utilizing ultrasound might decrease the risk of inadvertent vascular puncture. Significant bleeding and severe complications due to vascular puncture are uncommon.^9^

One major source of morbidity and mortality related to the use of local anesthetic is local anesthetic systemic toxicity (LAST). Although rare, the rising prevalence of local anesthetics in practice has resulted in a greater incidence of LAST, with bupivacaine-associated fatal cardiac toxicity presenting in higher numbers. LAST could result in mild systemic symptoms such as auditory changes, circumoral numbness, metallic taste, and agitation. LAST may also present more severely with complications related to the central nervous system (CNS) (i.e., seizure, coma, respiratory arrest) or cardiovascular system (i.e., hypertension, hypotension, tachycardia, bradycardia, ventricular arrhythmias, and cardiac arrest).^9^ The most common cause of LAST has been recognized to be due to unintentional intravascular injection during the administration of local anesthetics. Studies have shown varying reports on the incidence of LAST. Some studies report 0 events after over 12,000 nerve blocks; whereas others report an incidence of up to 25 per 10,000 nerve blocks. One study reported seizures in 79 of 10,000 brachial plexus blocks. Despite the inconsistency in these reports, it is notable to recognize that accidental intravascular injection during nerve blocks are related to LAST. Hence, UGRNBs can help in optimizing nerve block procedures to decrease the risk of intravascular injections, thus reducing the likelihood of nerve blocks that could cause LAST.^10^

Lipid emulsion therapy has been gaining support as a major treatment method for management of LAST. Past studies have reported on lipid infusion reversing LAST from ropivacaine and levobupivacaine. Recent studies further provide support for successful lipid infusion treatment of LAST and its contribution to improvement of cardiac conduction, contractility, and coronary perfusion by drawing the lipid-soluble local anesthetic out of the cardiac tissue.^9^, ^10^

Another advantage of UGRNBs is that they are commonly used to avoid alternative forms of anesthesia and analgesics such as general anesthesia and opioids, respectively. This is particularly important for patients who may be unable to tolerate the respiratory risks involved with these alternatives. Furthermore, it is imperative to minimize the risk of opioid-dependence whenever possible. Emergency departments have seen an upward trend of opioid-related visits and, according to the Centers for Disease Control and Prevention, there were 70,630 deaths attributable to opioid use in 2019 alone.^11^ Therefore, it has become vital for emergency medicine healthcare providers to be trained on strategies to reduce opioid administration. UGRNBs often achieve the analgesic goals otherwise accomplished by narcotics while avoiding many of the disadvantages.

The primary goal of this small-group workshop was to facilitate an environment for emergency medicine residents to sharpen their nerve blocking skills and develop the confidence needed to incorporate the technique into their patient management. This format was selected due to the emphasis on practical application of the skill being learned. Limited time was dedicated towards non-dexterity related knowledge such as dosing, indications/contraindication, and guidelines. Substantial time was focused on the physical skill of performing the nerve blocks. This included proper placement of the patient’s body and ultrasound probe, identification of anatomical landmarks, and needle insertion and injection of the anesthetic medication.

Among the residents, there was a variable range of prior knowledge and experience administering UGRNBs. The narrow-focused learning strategy resulted in an efficient approach that became an introduction into nerve blocks for first-year residents and a valuable refresher activity for older residents. Instructors encouraged participants to assist one another to maximize time spent. This format was designed to facilitate group learning and provide a medium for which those with more experience could share their knowledge. This was especially evident during the standardized patient and cadaver portions of the activity. Additionally, when one participant became more comfortable with the skill, they could challenge themselves by teaching the learners around them. Residents were observed discussing various tips and tricks such as ultrasound probe angles, how to position oneself to limit neck straining, where to keep one’s hands and arms for maximum stability, the importance of keeping one’s eyes on the ultrasound monitor, and the initial anatomical landmarks to search for.

Studies have described the benefits of using simulation-based learning strategies over more traditional learning methods. Simulation allows learners to take advantage of experiential learning modalities via artificial representation of true clinical scenarios. Deliberate practice of clinical skills through these “high-fidelity” approaches has been shown to provide a superior and more efficient environment for skill-learning. In part, this has been attributed to the ability of the learner to make mistakes with decreased fear of harming the patient.^12^ Due to the short timeframe and the evidence provided by previous studies, emphasis was placed on proper technique and multiple repetitions of the skills being taught. The result was an activity that appeared to effectively facilitate skill learning and was enjoyable for participants.

### Recommended pre-reading for facilitator

- Each UGRNB can be reviewed on the New York School of Regional Anesthesia (NYSORA) website:
  - Ultrasound-Guided Supraclavicular Brachial Plexus Nerve Block^1^
  - Ultrasound-Guided Infraclavicular Brachial Plexus Nerve Block^2^
  - Ultrasound-Guided Fascia Iliaca Nerve Block^3^
  - Ultrasound-Guided Femoral Nerve Block^4^
  - Ultrasound-Guided Popliteal Sciatic Nerve Block^5^
- Be familiar with the updated UGRNB policy statement by the American College of Emergency Physicians.^6^

### Learner responsible content (LRC)

- None

### Required Materials

Ultrasound Station:

- Standardized patients (preferably one per nerve block).
- Ultrasound machine with associated materials.
- Computer with the preloaded reference videos and guides.^13^–^19^

Cadaver Station:

- Cadaver with associated materials (preferably 2–3 students per cadaver).
- Cadaver alternative: Regional anesthesia simulation devices (For example: Simulab’s Regional Anesthesia Femoral Trainer)
- Nerve block kit (For example: Halyard ON-Q Pain Relief System, T-Bloc continuous echogenic nerve block tray).
- Additional echogenic needles (For example: Halyard Echobright echogenic single shot Tuohy needle 18 GA × 100 mm).
- Computer with the preloaded reference videos and guides.^13^–^19^

### Results and tips for successful implementation

This activity took place at a medical simulation center that had ultrasound machines, nerve block kits, and cadavers available. To aid in the effectiveness of learning during this workshop, we prioritized the presence of highly experienced instruction through guidance by an anesthesiologist with over 30 years of regional nerve block experience.

Emergency medicine residents alternated between two stations: a standardized patient ultrasound station and a cadaver station. Time spent at each station depended on the number of learners and abundancy of resources. The goal was to dedicate roughly 10–20 minutes per individual nerve block, resulting in 1.5 hour per station. These stations had computers with preloaded guides and videos for learners to reference during their time with each patient. In addition, these stations were encompassed by a 30-minute introductory presentation and a debriefing session. With an additional 15 minutes of buffer time for transitions and a break between stations, the total workshop time was roughly 4 hours.

The introductory presentation (Appendix A: UGRNB Introductory Presentation) reviewed common indications for regional nerve blocks, the benefits of using ultrasound, why UGRNBs may be preferred over other pain controlling modalities, tips for the specific blocks covered in the workshop, possible complications to be aware of, commonly used local anesthetics, and tips for a productive learning session.

This presentation was given by an experienced anesthesiologist. However, if limited by availability, this workshop could also be facilitated by an emergency medicine attending physician with UGRNB experience. Following this presentation, the group of residents divided into two groups. One group went to the ultrasound station while the other went to the cadaver station.

The ultrasound station included standardized patients. There were five total standardized patients, one for each block being learned during this activity (supraclavicular brachial plexus, infraclavicular brachial plexus, femoral, fascia iliaca, and popliteal fossa). The instructors hovered around each of the patients to facilitate questions from learners, in addition to assisting with anatomical landmark identification and proper patient/probe positioning. Residents were at the bedside of each standardized patient and were asked to identify the relevant anatomical structures with the ultrasound probe. This station gave residents the opportunity to apply their medical knowledge and to build foundations for the basics of ultrasound in a comfortable setting alongside the guidance of their instructors. This station was valuable for learners to become familiar with and quickly recognize important anatomical landmarks on ultrasound with specific regard to nerve blocks.

The cadaver station was focused on hand-eye coordination with needle and probe in hand, giving residents an opportunity to simulate performing the various blocks and sharpen their technique. Although the station provided a valuable learning experience, there were some notable disadvantages. Cadaver tissue is often stiffer than live tissue and vessels may have collapsed due to lack of blood flow. For this reason, it was helpful to have recently embalmed cadavers. Additionally, fluid was not actually injected as that would distort anatomical landmarks for subsequent learners using the same cadaver. As the workshop progressed, tissue damage naturally emerged at the sites being practiced on. This was circumnavigated to a degree by limiting the first group to using the right side of cadaver 1 and the left side of cadaver 2. The second group was then able to use the opposite sides, providing them the opportunity to practice on undamaged tissue. This station allowed residents to improve their needle-probe coordination techniques in a safe and supervised environment.[Fig f6-jetem-7-3-sg24][Fig f7-jetem-7-3-sg24][Fig f8-jetem-7-3-sg24][Fig f1-jetem-7-3-sg24][Fig f2-jetem-7-3-sg24][Fig f3-jetem-7-3-sg24][Fig f4-jetem-7-3-sg24][Fig f5-jetem-7-3-sg24]

Following the two main stations, the group gathered for a debriefing session (Appendix B: UGRNB Debrief Instructor Guide). The goal of this session was to answer any questions not previously addressed, review what was learned, and provide resources for additional learning. This session was organized by two main parts:

Part 1: Reflection and analysis of the activity

- Any comments about how you feel the activity went?
- What is something you struggled with at the beginning that you feel more comfortable with now?
- Was there any adjustment you made during the session that made a certain block easier to perform? (eg, body position, hand placement, certain landmarks, movements with the transducer, etc.)
- Learning objectives:
  ○ Can you name some clinical situations where UGRNBs may be used?
  ○ Can you name some risks you face when doing nerve blocks?
  ○ Can you name some steps you may take when performing a nerve block?

Part 2: Reminders and take-aways:

- Order of events: patient position, personal position, probe position, identify landmarks, needle insertion with eyes on the monitor, aspirate before injection, monitor the injection.
- Perform each block the exact same way every time regardless of the situation; then work on increasing speed without compromising safety.
- Make ergonomic adjustments (eg, increasing bed height to avoid causing discomfort or instability during the procedure).
- Compressing structures may make it easier to identify landmarks like arteries.
- Movements with needle and probe should be subtle.
- Always keep eyes on the monitor to avoid losing coordination and position of the needle head.

Following the two main portions of the debriefing session, learners are given an opportunity to ask any other questions they may have. Lastly, this session elicited feedback from the learners (Appendix C: UGRNB Evaluation Survey). During the pilot run of this workshop, feedback was gathered verbally; however, in future workshops, learners will be asked to fill out an anonymous evaluation addressing various aspects of the activity and future improvements to be made. An example of this survey asks learners to agree or disagree with the following statements on a numerical scale where 5 indicates *strongly agree*, 3 is *neutral*, and 1 indicates *strongly disagree*:

- The introductory presentation provided at the beginning of this workshop was informative and clearly outlined the expectations.
- The information provided in this workshop contributed to my learning.
- Clinical faculty in this workshop provided effective teaching.
- This workshop provided positive learning environment, eg, adequate equipment, involvement of staff.
- I was given adequate time at each station during the workshop.
- The workshop was organized and was not difficult to follow.
- I was given appropriate independence during the workshop, eg, allowed to practice skills on own.
- Instruction was tailored to my needs and current level of competence.
- Compared to prior to completing this workshop, I feel more confident with using ultrasound after completing this workshop.
- Compared to prior to completing this workshop, I feel more confident performing nerve blocks after completing this workshop.
- Please provide an overall grade for this workshop. (4-Excellent; 3-Good; 2-Fair; 1-Poor)

After completing the quantitative portion of the survey, a comment section asks learners to provide one positive and one negative aspect of this workshop. When feedback was gathered verbally, learners were asked questions such as:

- What is something valuable you learned today?
- Did you find the workshop useful to your growth as a clinician?
- Do you feel more comfortable performing UGRNBs?
- Do you have any constructive feedback or ideas to improve this workshop in the future?
- Was there anything that wasn’t covered that you wish had been?

All learners agreed that the workshop was a valuable activity and that they felt more comfortable performing UGRNBs. Some learners expressed appreciation for the pragmatically focused nature of the activity. One learner commented that they would like to have seen an additional cadaver available due to the ratio of learners to cadavers being small and feeling congested. Another learner stated that they would have liked the standardized patients to have a wider range of body habitus or age to provide greater variability and diversify anatomical landmark identification. Lastly, a learner commented that the cadaver station felt slightly rushed while the ultrasound station could have been done in less time.

If the availability of resources permits, providing greater variability in standardized patients and an increased number of cadavers should be considered. It may be helpful to switch the format of the workshop from two stations to a free-roam format, allowing learners to direct their focus to skills they would like to improve while devoting less time to skills they already feel comfortable with. Considering the range of prior experience, groups may be comprised of an equal number of participants from each skill level. For example, an equal number of interns and PGY-2/3/4s should be added to each group. These adjustments provide a more efficient approach for learners to strengthen their techniques while maintaining the learning goals and objectives of the workshop.

### Pearls

This is included separately as Appendix B, “Ultrasound-Guided Regional Nerve Block Pearls” Word document, which can be printed out and distributed as a handout after the course.)

## Figures and Tables

**Figure 1 f1-jetem-7-3-sg24:**
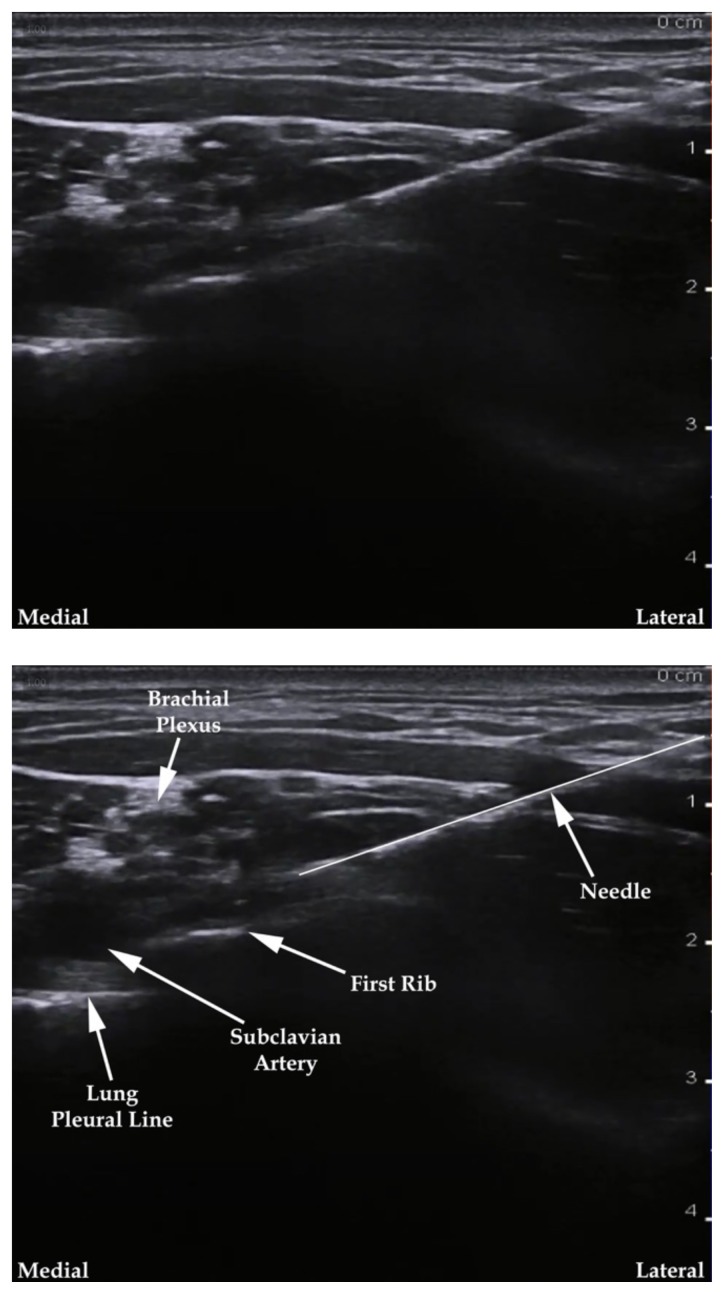
Ultrasound-guided supraclavicular brachial plexus nerve block. Modified from source.^13^

**Figure 2 f2-jetem-7-3-sg24:**
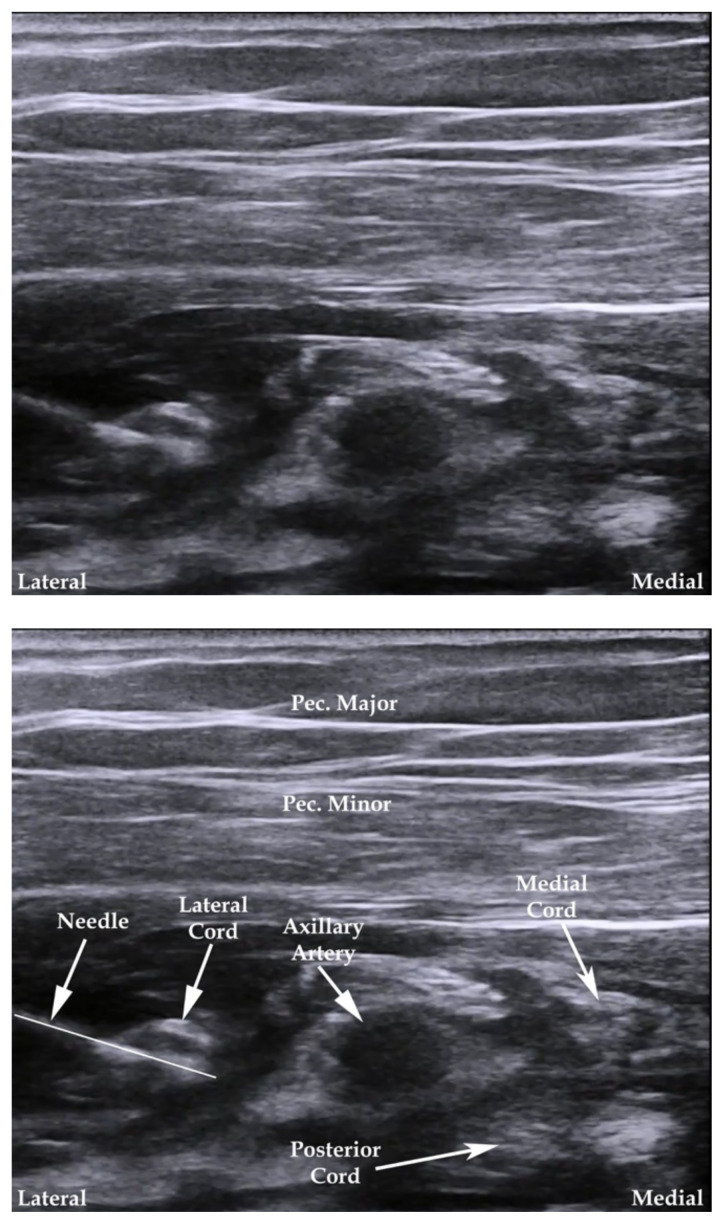
Ultrasound-guided infraclavicular brachial plexus nerve block. Modified from source.^14^

**Figure 3 f3-jetem-7-3-sg24:**
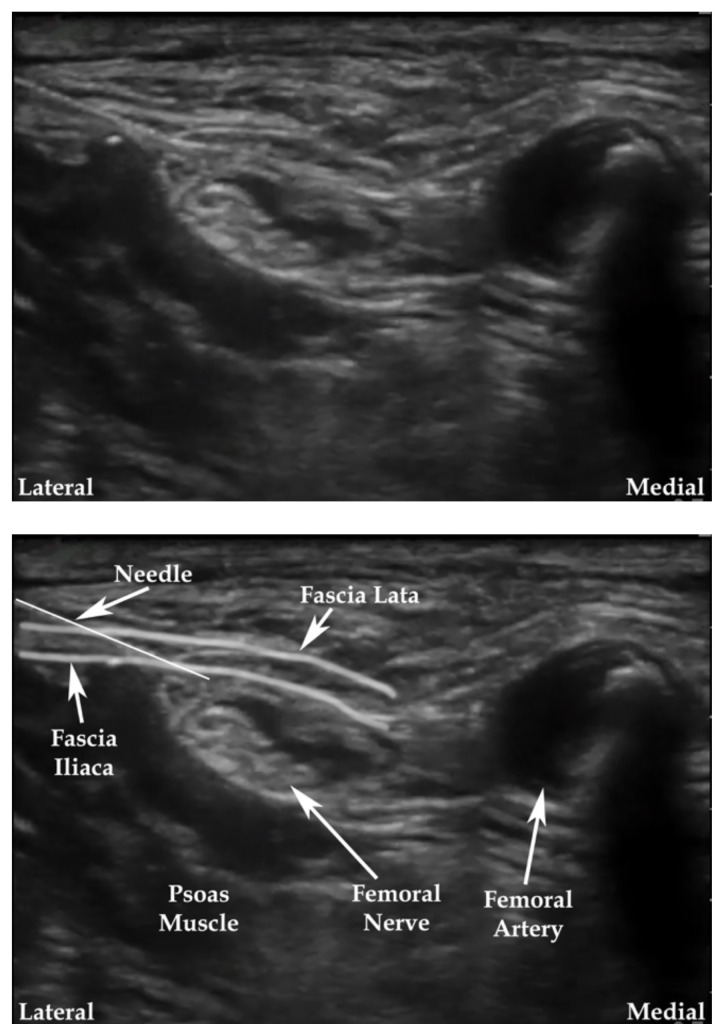
Ultrasound-guided femoral nerve block. Modified from source.^15^

**Figure 4 f4-jetem-7-3-sg24:**
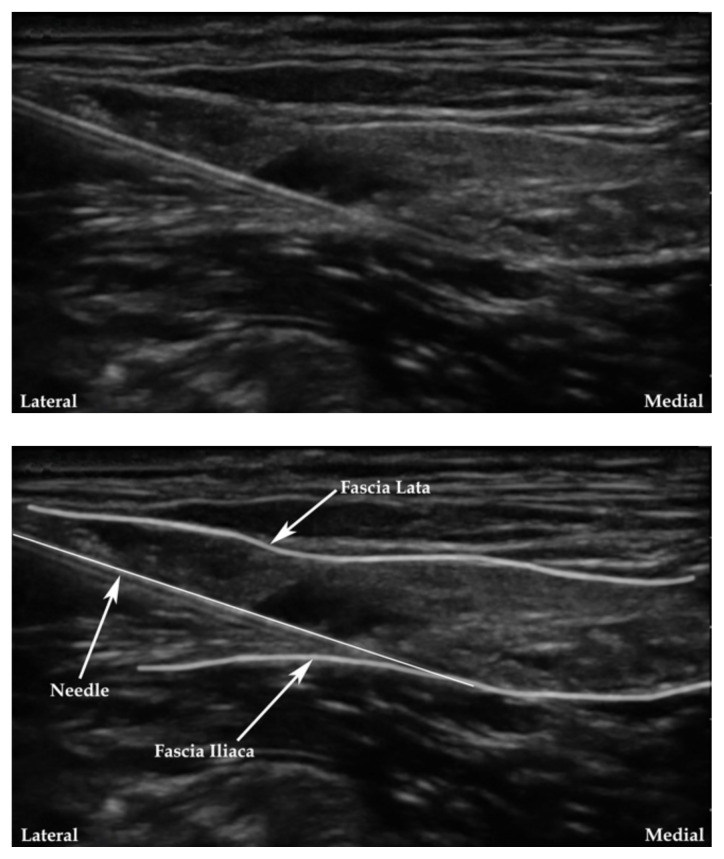
Ultrasound-guided fascia iliaca nerve block. Modified from source.^16^

**Figure 5 f5-jetem-7-3-sg24:**
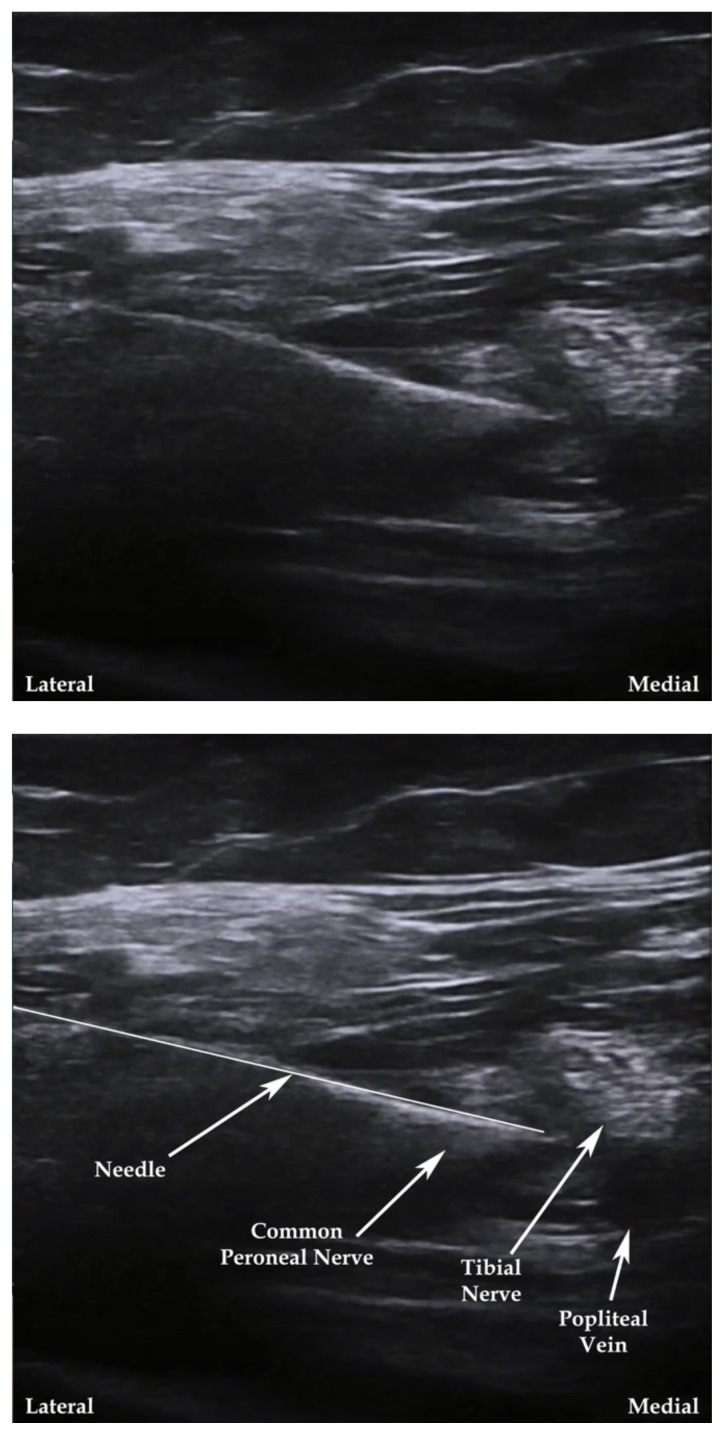
Ultrasound-guided popliteal fossa nerve block. Modified from source.^17^

**Image 1 f6-jetem-7-3-sg24:**
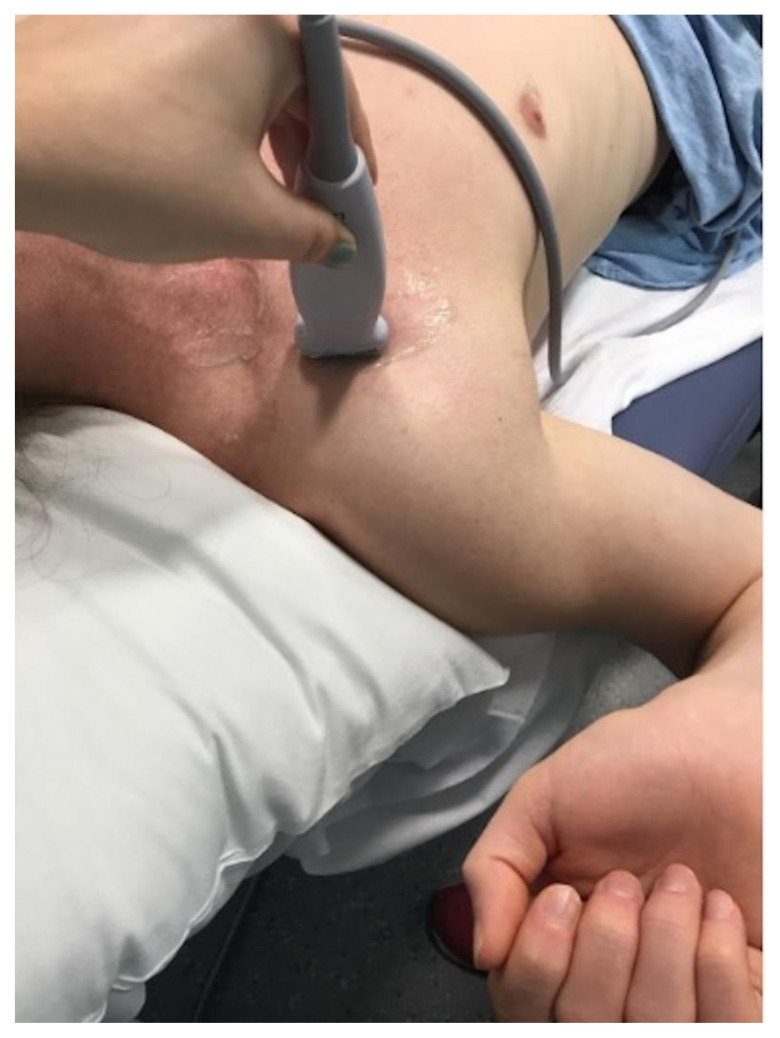
Supraclavicular Brachial Plexus Block. Author’s own image.

**Image 2 f7-jetem-7-3-sg24:**
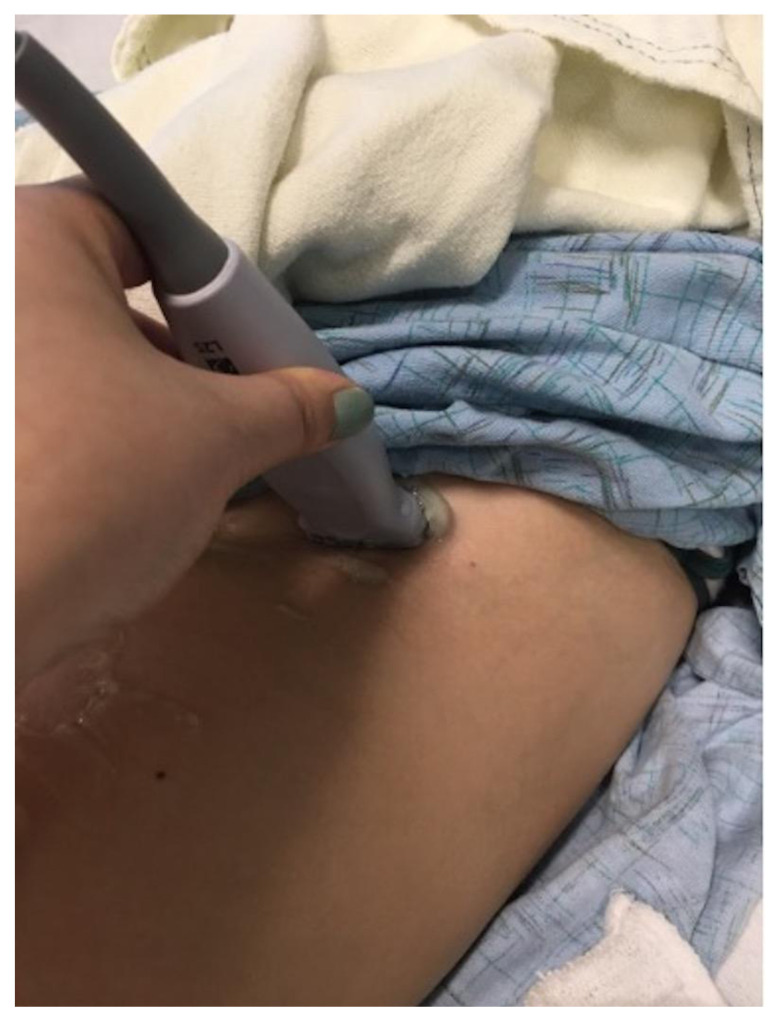
Femoral Block. Author’s own image.

**Image 3 f8-jetem-7-3-sg24:**
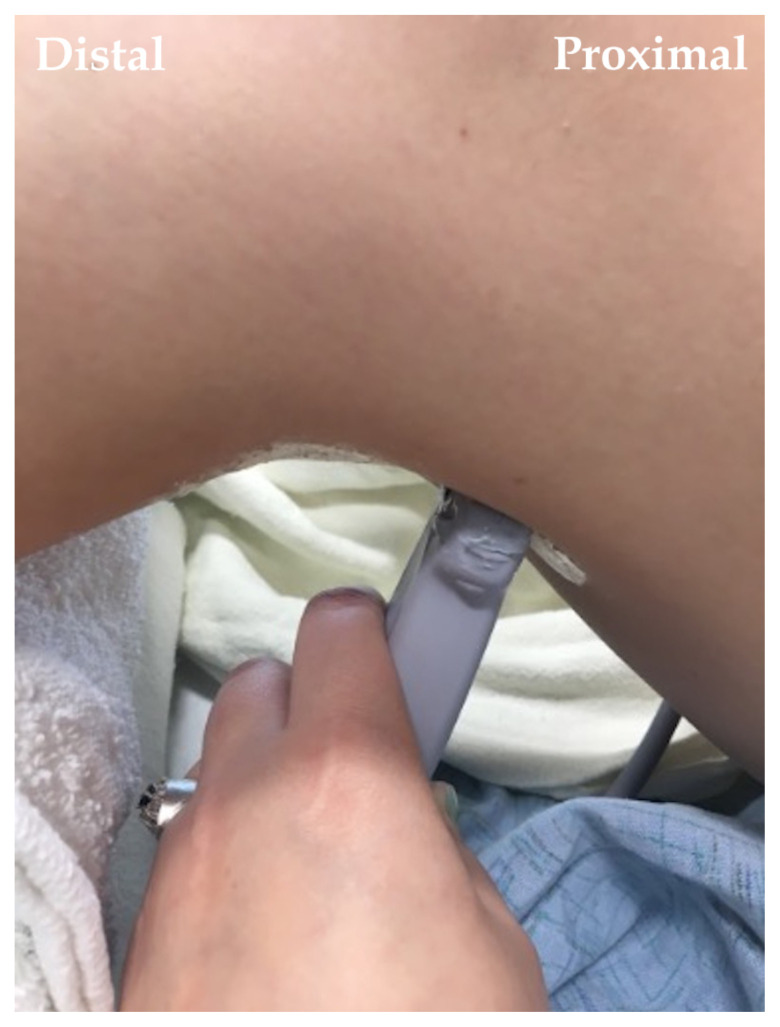
Popliteal Fossa Block. Author’s own image.

**Image 1:**
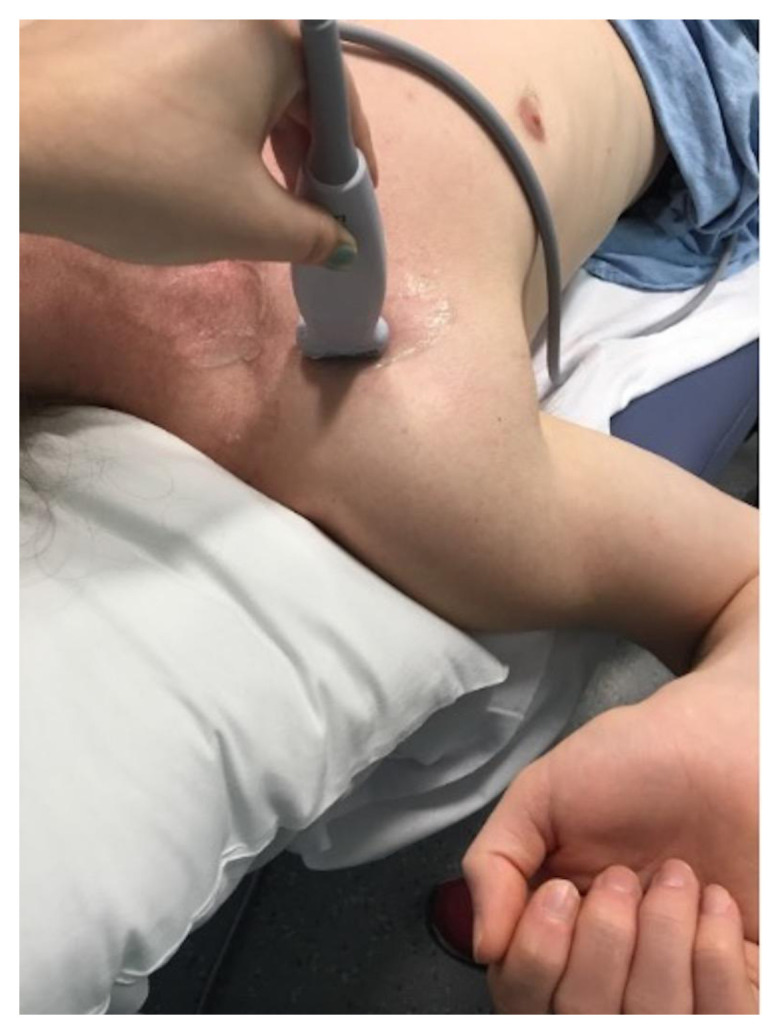
Supraclavicular Brachial Plexus Block. Author’s own image.

**Figure 1:**
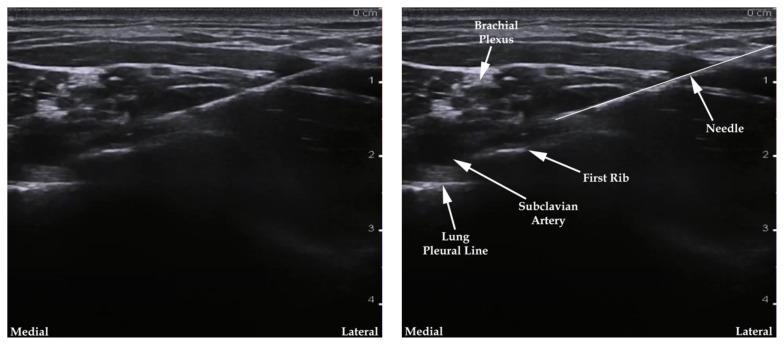
Ultrasound-guided supraclavicular brachial plexus nerve block. Modified from source.^13^

**Figure 2:**
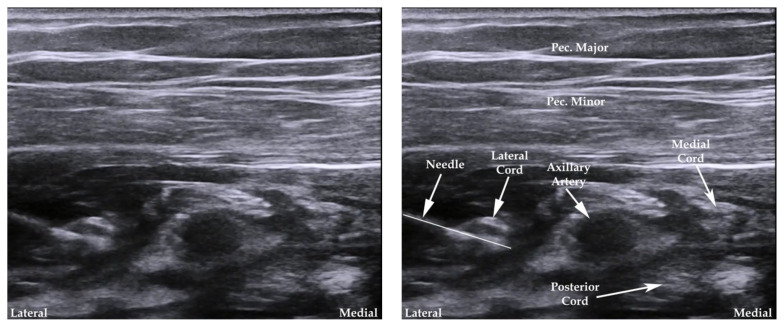
Ultrasound-guided infraclavicular brachial plexus nerve block. Modified from source.^14^

**Image 2:**
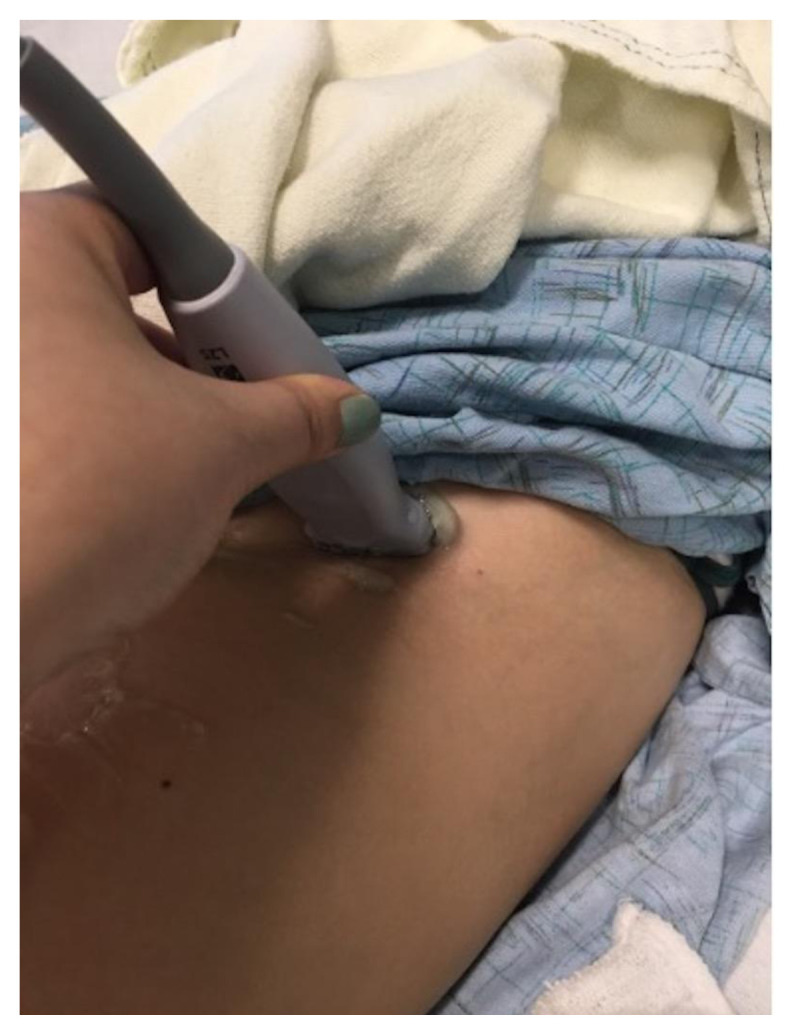
Femoral Block. Author’s own image.

**Figure 3:**
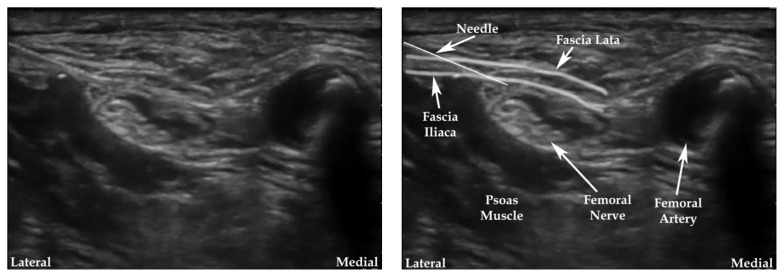
Ultrasound-guided femoral nerve block. Modified from source.^15^

**Figure 4:**
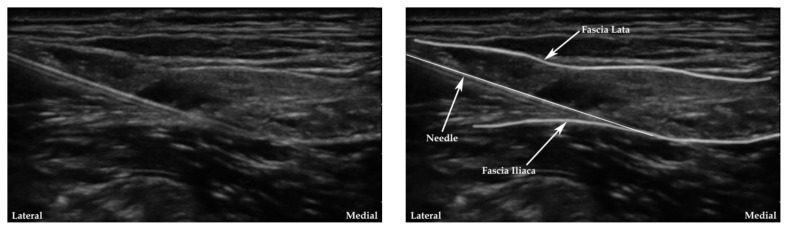
Ultrasound-guided fascia iliaca nerve block. Modified from source.^16^

**Image 3:**
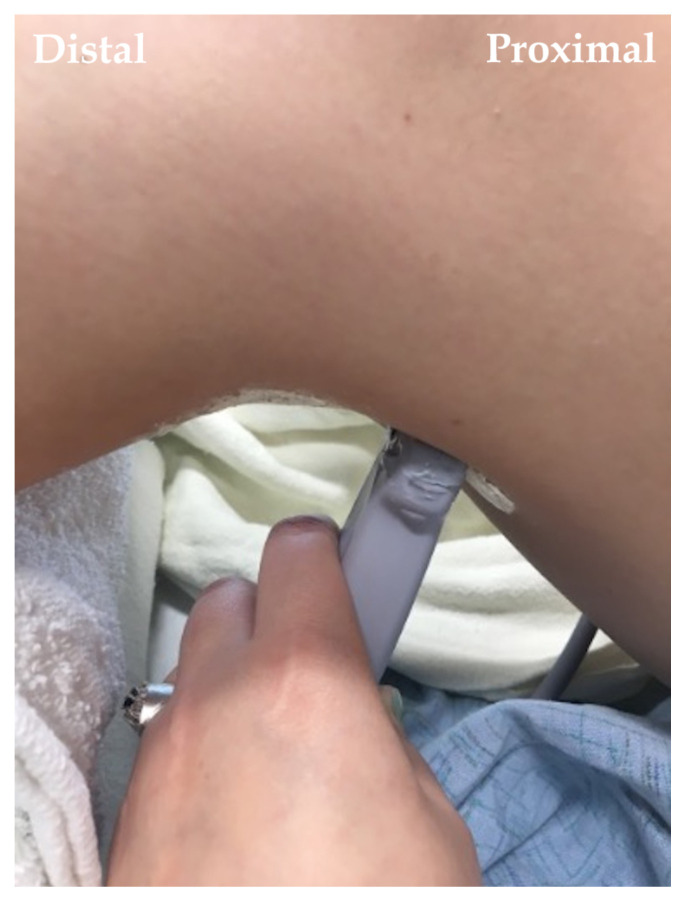
Popliteal Fossa Block. Author’s own image.

**Figure 5:**
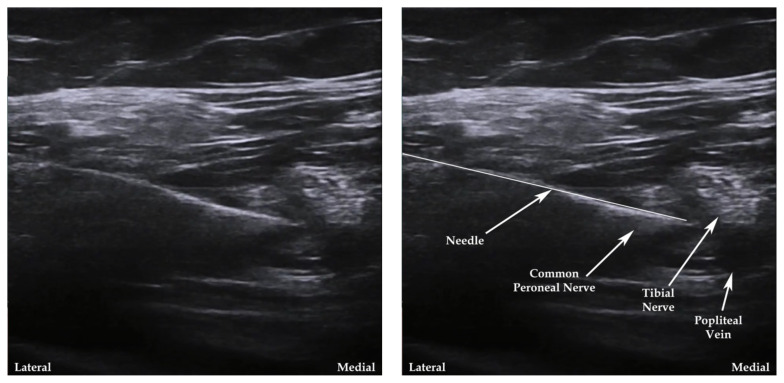
Ultrasound-guided popliteal fossa nerve block. Modified from source.^17^

## SMALL GROUPS LEARNING MATERIALS

### Appendix A Ultrasound-Guided Regional Nerve Block Introductory Presentation

#### Supplementary Information

PowerPoint file.

### Appendix B Ultrasound-Guided Regional Nerve Block Pearls

#### Safety

- *Linked objective:* Recognize the clinical situations in which UGRNBs can be utilized and understand the associated risks.
- To minimize room for mistakes, perform each respective block the exact same way each time it is done regardless of the situation.
- Make ergonomic adjustments such as increasing bed height to avoid causing discomfort or instability during the procedure.
- Recognize the various clinical signs and symptoms of local anesthetic systemic toxicity (LAST).

#### Anesthetic Medications^18^

- *Linked objective:* List the commonly used local anesthetic medications and their proper dosing in respect to regional nerve blocks.

**Table 1:** Commonly used local anesthetics for peripheral nerve blocks^19^, ^20^

Local Anesthetic	Concentration (%)	Onset (min)	Duration (h)	Max Dose (mg/kg)	Max Total Dose (mg)
Lidocaine	1.5–2	10–20	2–5	4.5	300 (15 ml of 2%)
Mepivacaine	1.5–2	10–20	2–5	5	400 (20 ml of 2%)
Bupivacaine	0.25–0.5	15–30	10–14	2.5	175 (70 ml of 0.25%)
Ropivacaine	0.5–1	15–30	4–12	3	225 (45 ml of 0.5%)

#### Ultrasound Positioning

- *Linked objective:* Demonstrate proper ultrasound probe positioning and identify relevant anatomical landmarks for each nerve block on both standardized patients and cadavers.
- First place patients in the proper position.
- Compressing structures such as vessels can make them easier to find.
- Always identify anatomical landmarks prior to needle insertion.

#### Needle-Probe Technique

- *Linked objective:* Describe the common steps involved to perform each nerve block.
- Position the patient appropriately and then adjust oneself to avoid strain or instability during the proceeding.
- Identify anatomical landmarks with the ultrasound.
- Place the needle in-plane with the probe and keep eyes on the monitor to avoid losing position and coordination.
- Movements with the needle and probe should be subtle.
- Movement of the skin will result in the needle moving (For example, if one stops applying forward pressure on the needle the skin retracts and pulls the needle backwards with it.)
- Always aspirate prior to injecting to prevent injecting into vessels.
- Monitor injection of the anesthetic on the ultrasound to confirm appropriate location and distribution.

#### Individual Block Tips

- *Linked objective:* Perform the five UGRNB techniques outlined in this workshop.
- Supraclavicular Brachial Plexus
  ○ Patient: Any position that is comfortable without blocking the needle and probe is acceptable.
  ○ Probe: Place in the transverse plane proximal to the clavicle and tilt caudally to view cross-section of the subclavian artery. Brachial plexus is seen superficial to the subclavian artery.
  ○ Needle: Insert in plane from lateral to medial toward the brachial plexus
  ○ Helpful hint: Prioritize keeping the probe perpendicular to the subclavian artery while moving it up the patient’s neck from lateral to medial until brachial plexus is seen and positioned properly.
- Infraclavicular Brachial Plexus
  ○ Patient: Supine with head turned contralaterally to the block. Abduct the humerus to 90 degrees.
  ○ Probe: Place in parasagittal plane, medial to the coracoid process and inferior to the clavicle to identify the axillary artery.
  ○ Needle: Insert in-plane of the probe, just inferior to the clavicle, and aim toward the posterior aspect of the axillary artery until needle is beneath the artery.
  ○ Helpful hint: Avoid placing the probe medial of the parasagittal plane.
- Femoral
  ○ Patient: Supine with the ipsilateral extremity abducted to roughly 15 degrees and externally rotated as to rest the lateral aspect of the foot on the exam table.
  ○ Probe: Place transversely on the femoral crease, over the femoral arterial pulse, and move medially to identify the artery.
  ○ Needle: Place adjacent to the lateral aspect of the femoral nerve and insert between the layers of the fascia iliaca that surround the femoral nerve.
  ○ Helpful hint: Identify the femur prior to the femoral artery.
- Fascia Iliaca
  ○ Patient: Supine position
  ○ Probe: Place transversely over the inguinal crease and move around slowly to identify the femoral artery, then move laterally to identify the femoral nerve followed by the fascia iliaca.
  ○ Needle: Insert in-plane from lateral aspect and guide the tip to under the fascia iliaca several centimeters lateral to the femoral artery.
  ○ Helpful hint: Confirm adequate spread of the anesthetic underneath the fascia iliaca with the ultrasound.
- Popliteal Fossa
  ○ Patient: Supine with knee flexed to 45–90 degrees
  ○ Probe: Place on the posterior aspect of knee to identify the sciatic nerve.
  ○ Needle: Insert in-plane from the lateral aspect to place the tip within the common sciatic nerve sheath.
  ○ Helpful hint: Identify the kidney shaped sheath surrounding the tibial and common fibular nerves and place the needle directly in-between.

### Appendix C Ultrasound-Guided Regional Nerve Block Debrief Instructor Guide

#### Reflection and analysis of the activity

1. Any comments about how you feel the activity went?
2. What is something you struggled with at the beginning that you feel more comfortable with now?
3. Was there any adjustment you made during the session that made a certain block easier to perform? (eg, body position, hand placement, certain landmarks, movements with the transducer, etc.)
4. Learning objectives:
  - Can you name some clinical situations where UGRNBs may be used?
  - Can you name some risks you face when doing nerve blocks?
  - Can you name some steps you may take when performing a nerve block?

#### Reminders and take-aways

1. Order of events: patient position, personal position, probe position, identify landmarks, needle insertion with eyes on the monitor, aspirate before injection, monitor the injection.
2. Perform each block the exact same way every time regardless of the situation; then work on increasing speed without compromising safety.
3. Make ergonomic adjustments (eg, increasing bed height to avoid causing discomfort or instability during the procedure).
4. Compressing structures may make it easier to identify landmarks like arteries.
5. Movements with needle and probe should be subtle.
6. Always keep eyes on the monitor to avoid losing coordination and position of the needle head.

**Address any questions learners may have**.

**Ask learners to fill out the feedback survey**.

### Appendix D Ultrasound-Guided Regional Nerve Block Post-Workshop Evaluation Survey

**Table:**

The introductory presentation provided at the beginning of this workshop was informative and clearly outlined the expectations.				
1-Stronly Disagree	2-Disagree	3-Neutral	4-Agree	5-Strongly Agree
The information provided in this workshop contributed to my learning.				
1-Stronly Disagree	2-Disagree	3-Neutral	4-Agree	5-Strongly Agree
Clinical faculty in this workshop provided effective teaching.				
1-Stronly Disagree	2-Disagree	3-Neutral	4-Agree	5-Strongly Agree
This workshop provided a positive learning environment, eg, adequate equipment, involvement of staff.				
1-Stronly Disagree	2-Disagree	3-Neutral	4-Agree	5-Strongly Agree
I was given adequate time at each station during the workshop.				
1-Stronly Disagree	2-Disagree	3-Neutral	4-Agree	5-Strongly Agree
The workshop was organized and was not difficult to follow.				
1-Stronly Disagree	2-Disagree	3-Neutral	4-Agree	5-Strongly Agree
I was given appropriate independence during the workshop, eg, allowed to practice skills on own.				
1-Stronly Disagree	2-Disagree	3-Neutral	4-Agree	5-Strongly Agree
Instruction was tailored to my needs and current level of competence.				
1-Stronly Disagree	2-Disagree	3-Neutral	4-Agree	5-Strongly Agree
Compared to prior to completing this workshop, I feel more confident with using ultrasound after completing this workshop.				
1-Stronly Disagree	2-Disagree	3-Neutral	4-Agree	5-Strongly Agree
Compared to prior to completing this workshop, I feel more confident performing nerve blocks after completing this workshop.				
1-Stronly Disagree	2-Disagree	3-Neutral	4-Agree	5-Strongly Agree
Please provide an overall grade for this workshop.				
1-Poor	2-Fair	3-Good	4-Excellent	

Please provide one positive aspect of this workshop:

Please provide one negative aspect of this workshop:

Any Other Comments:
