# Supplementary material for: High-Efficiency Ultrasound-Guided Regional Nerve Block Workshop for Emergency Medicine Residents
Source: J Educ Teach Emerg Med. 2022 Jul 15;7(3):SG24–44. doi: 10.21980/J84P8R (PMC10332701; doi:10.21980/J84P8R)
Supplement: Supplementary file 1 — PowerPoint file. [file jetem-7-3-sg24-appendixA.pptx]

## Slide 1
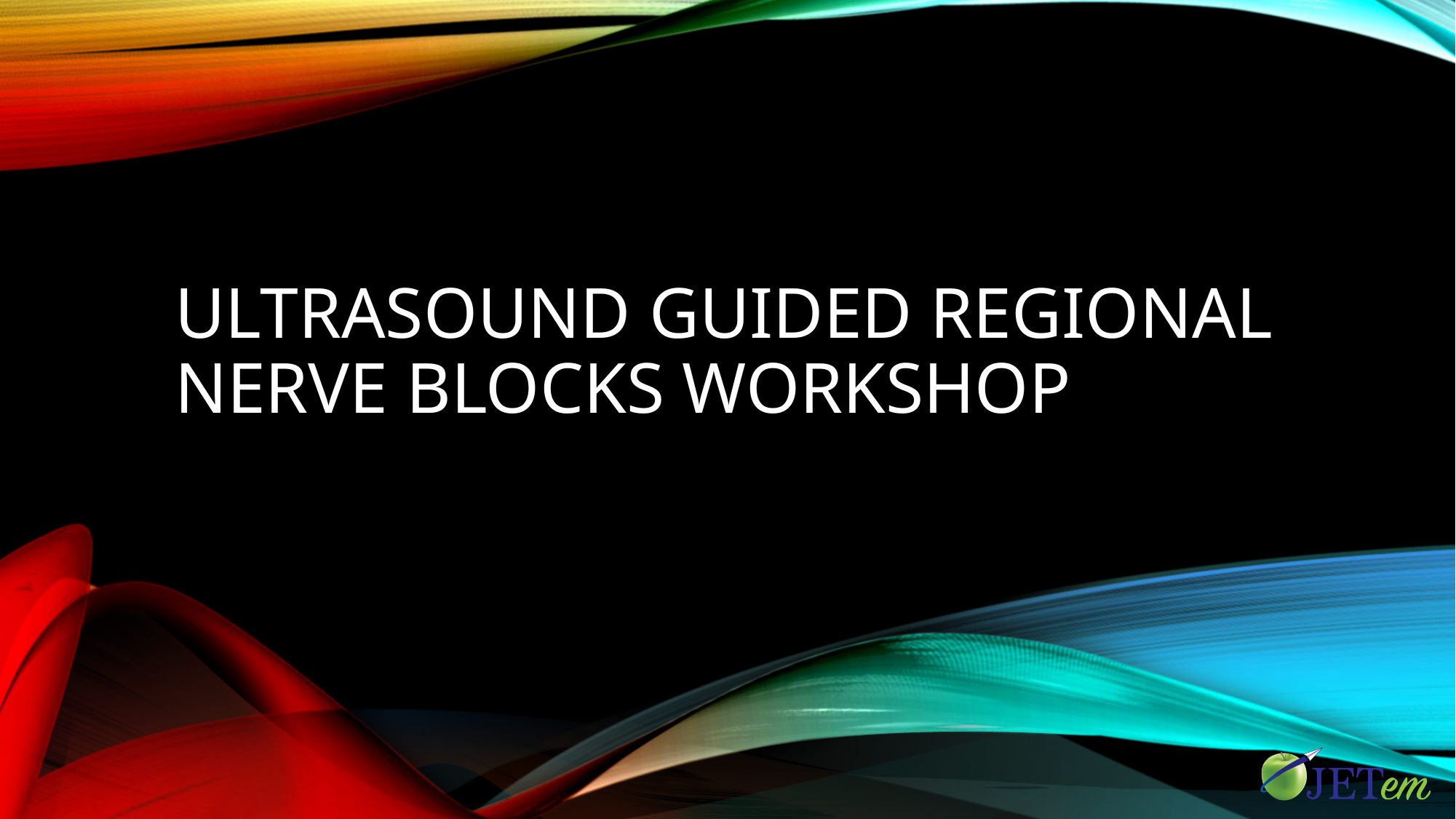

# Ultrasound Guided Regional Nerve Blocks Workshop

## Slide 2
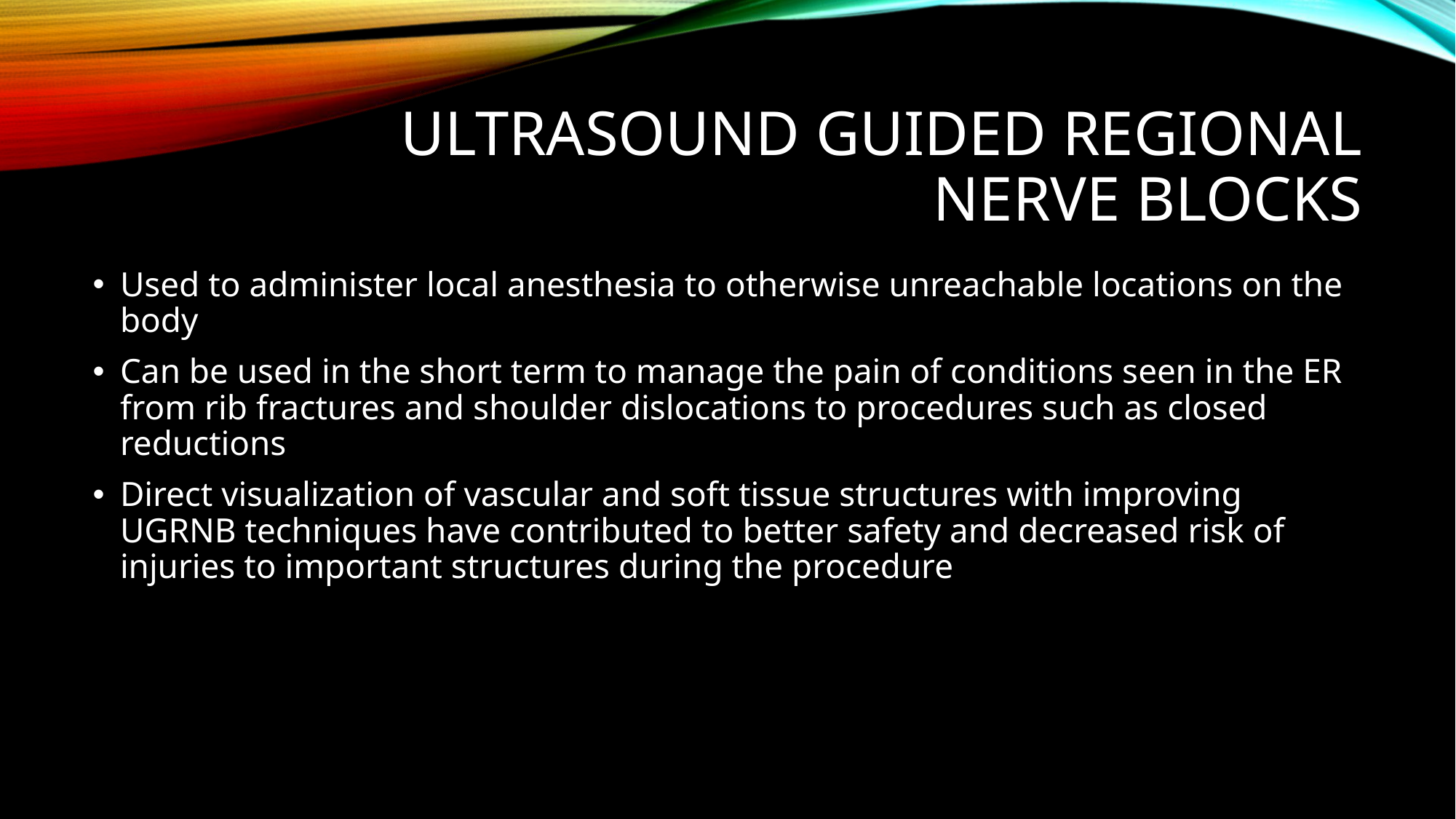

# Ultrasound Guided Regional Nerve Blocks
Used to administer local anesthesia to otherwise unreachable locations on the body
Can be used in the short term to manage the pain of conditions seen in the ER from rib fractures and shoulder dislocations to procedures such as closed reductions
Direct visualization of vascular and soft tissue structures with improving UGRNB techniques have contributed to better safety and decreased risk of injuries to important structures during the procedure

## Slide 3
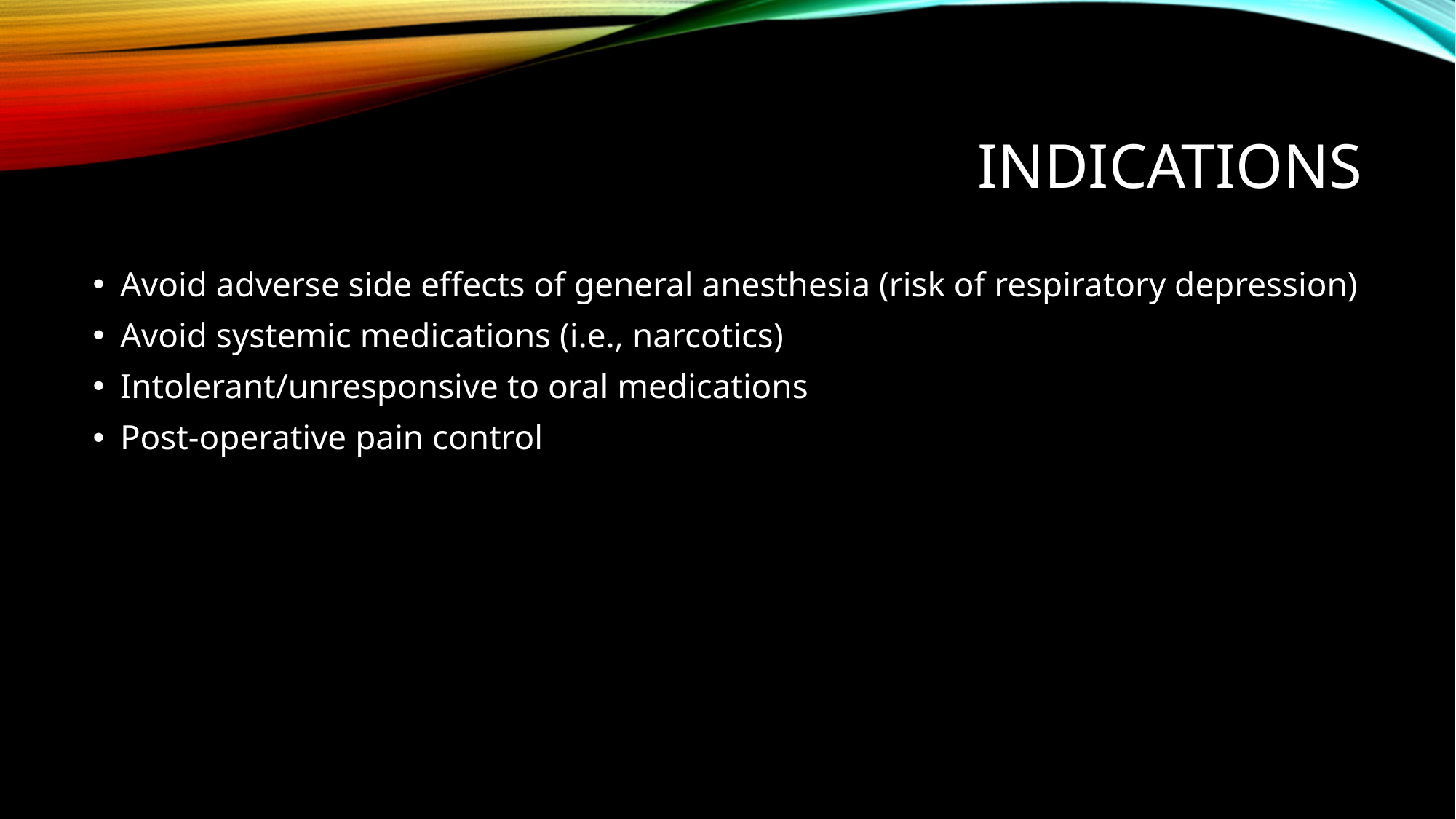

# Indications
Avoid adverse side effects of general anesthesia (risk of respiratory depression)
Avoid systemic medications (i.e., narcotics)
Intolerant/unresponsive to oral medications
Post-operative pain control

## Slide 4
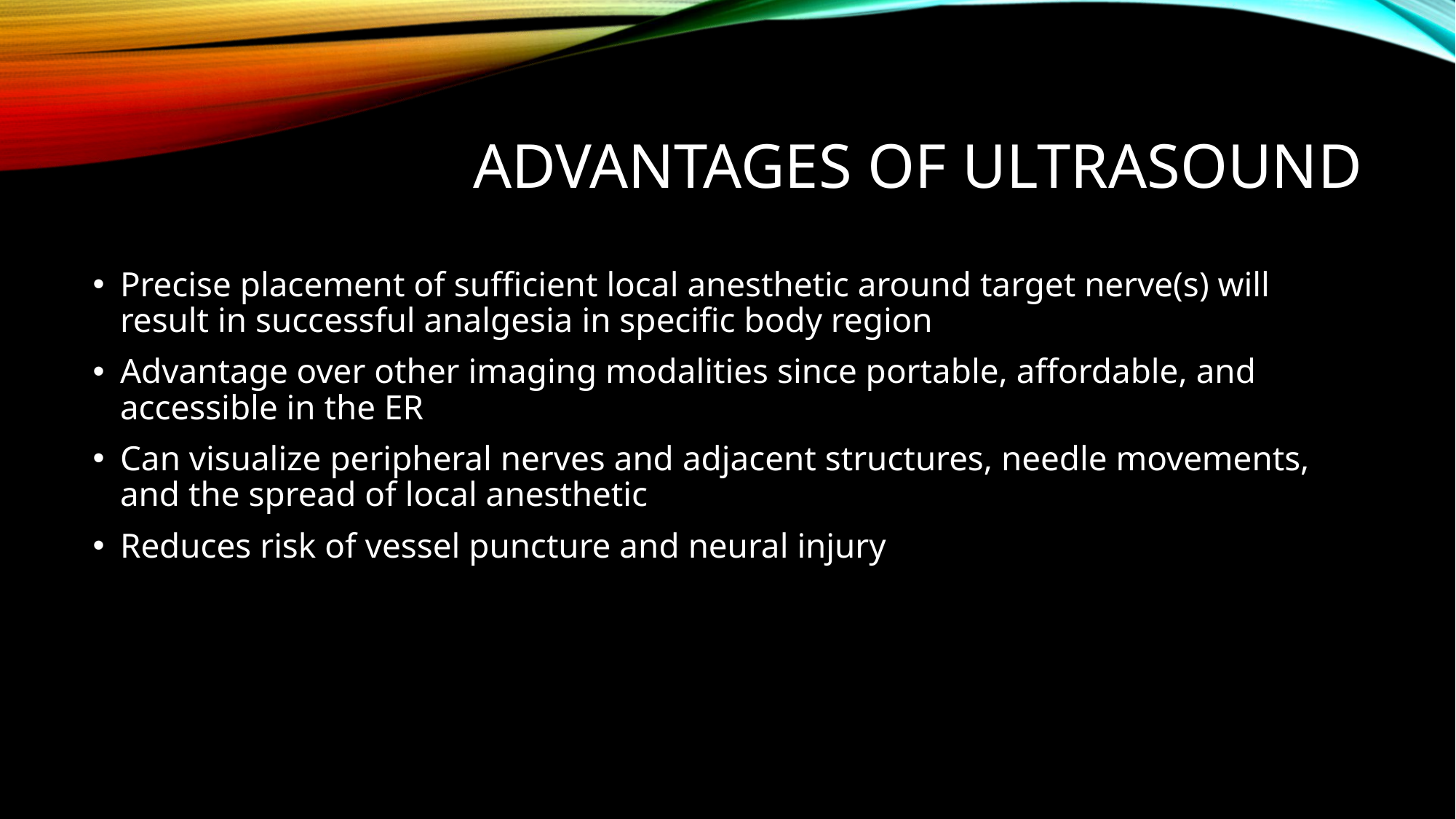

# Advantages of Ultrasound
Precise placement of sufficient local anesthetic around target nerve(s) will result in successful analgesia in specific body region
Advantage over other imaging modalities since portable, affordable, and accessible in the ER
Can visualize peripheral nerves and adjacent structures, needle movements, and the spread of local anesthetic
Reduces risk of vessel puncture and neural injury

## Slide 5
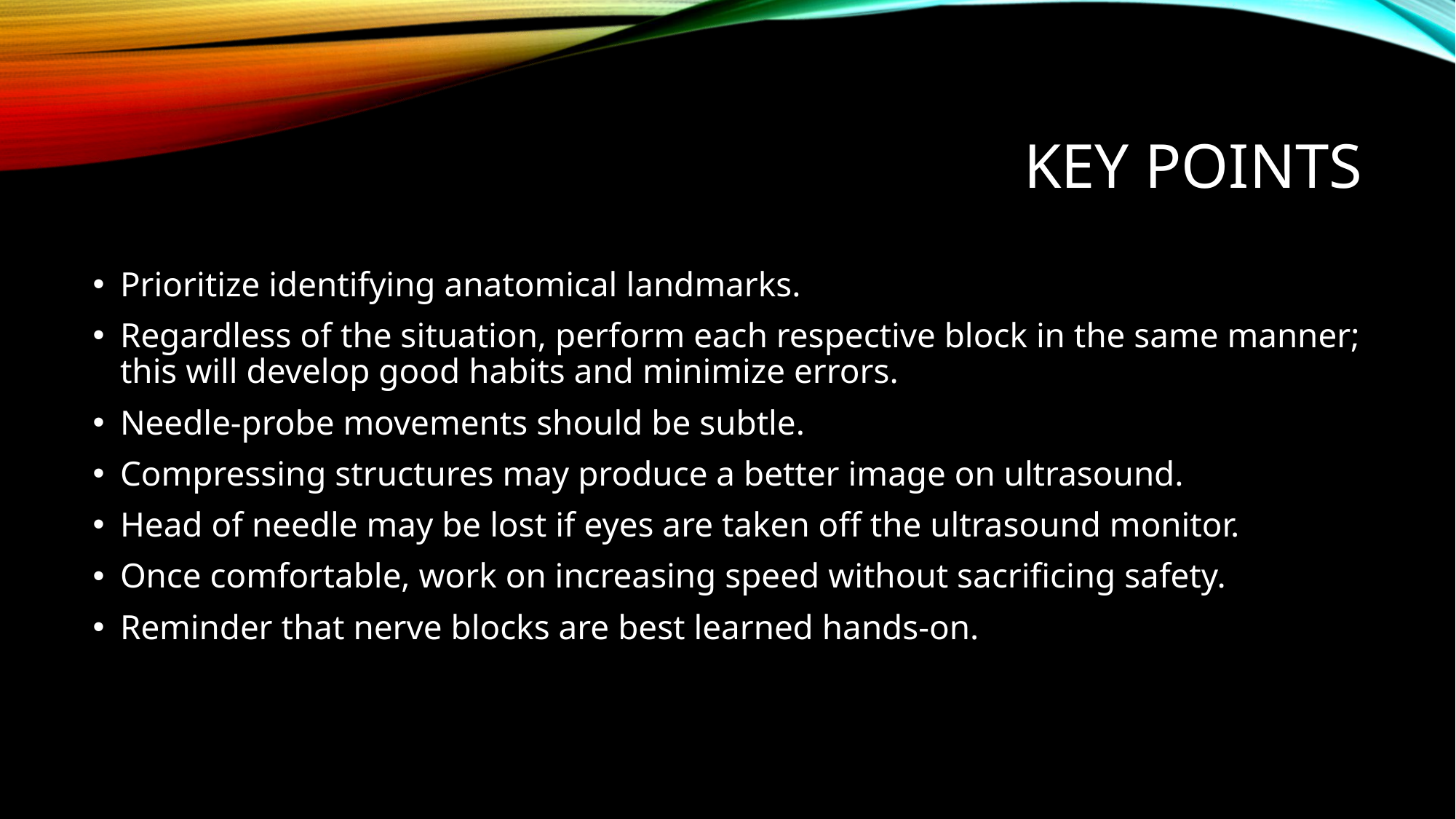

# Key Points
Prioritize identifying anatomical landmarks.
Regardless of the situation, perform each respective block in the same manner; this will develop good habits and minimize errors.
Needle-probe movements should be subtle.
Compressing structures may produce a better image on ultrasound.
Head of needle may be lost if eyes are taken off the ultrasound monitor.
Once comfortable, work on increasing speed without sacrificing safety.
Reminder that nerve blocks are best learned hands-on.

## Slide 6
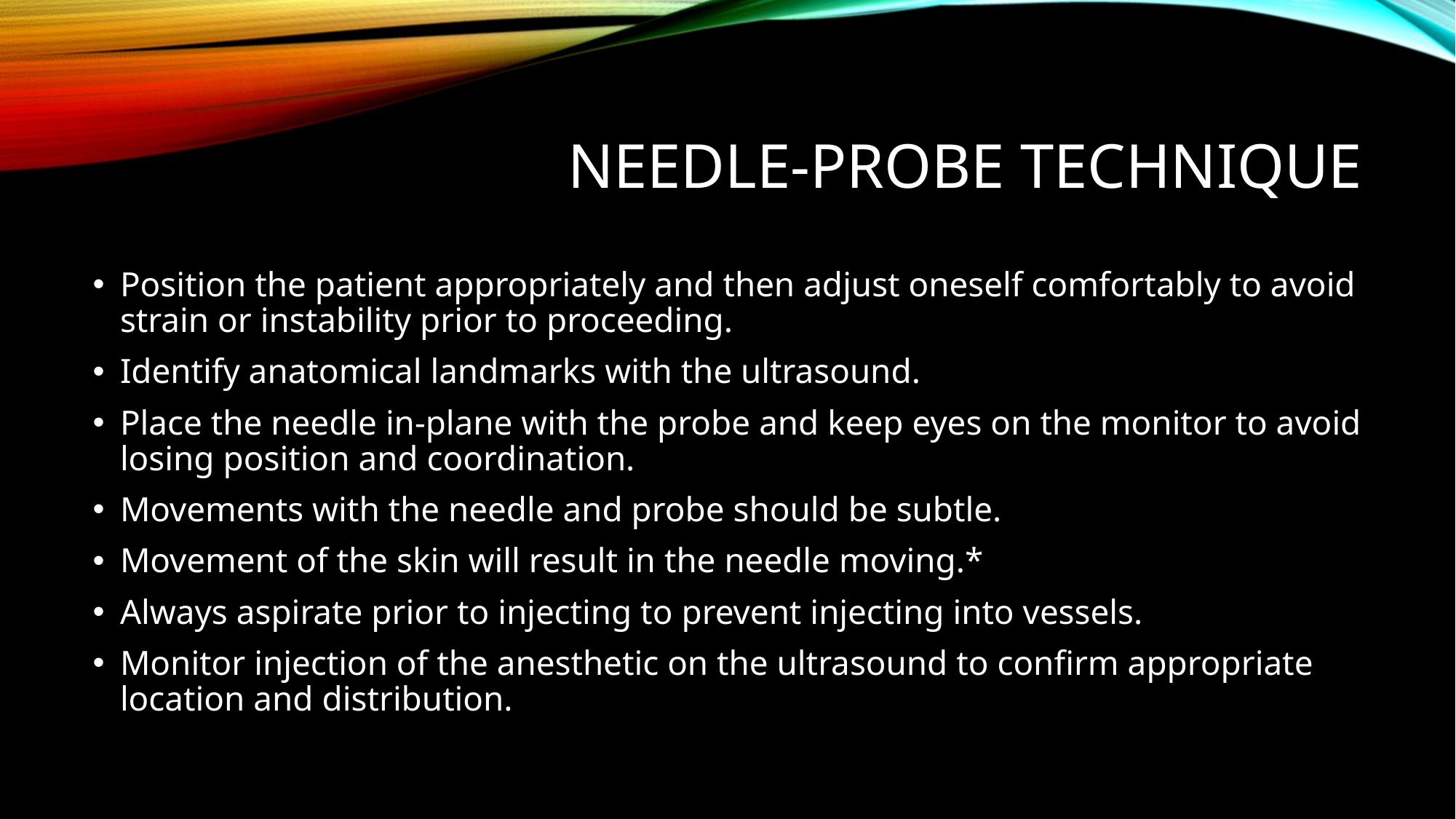

# Needle-Probe Technique
Position the patient appropriately and then adjust oneself comfortably to avoid strain or instability prior to proceeding.
Identify anatomical landmarks with the ultrasound.
Place the needle in-plane with the probe and keep eyes on the monitor to avoid losing position and coordination.
Movements with the needle and probe should be subtle.
Movement of the skin will result in the needle moving.*
Always aspirate prior to injecting to prevent injecting into vessels.
Monitor injection of the anesthetic on the ultrasound to confirm appropriate location and distribution.

## Slide 7
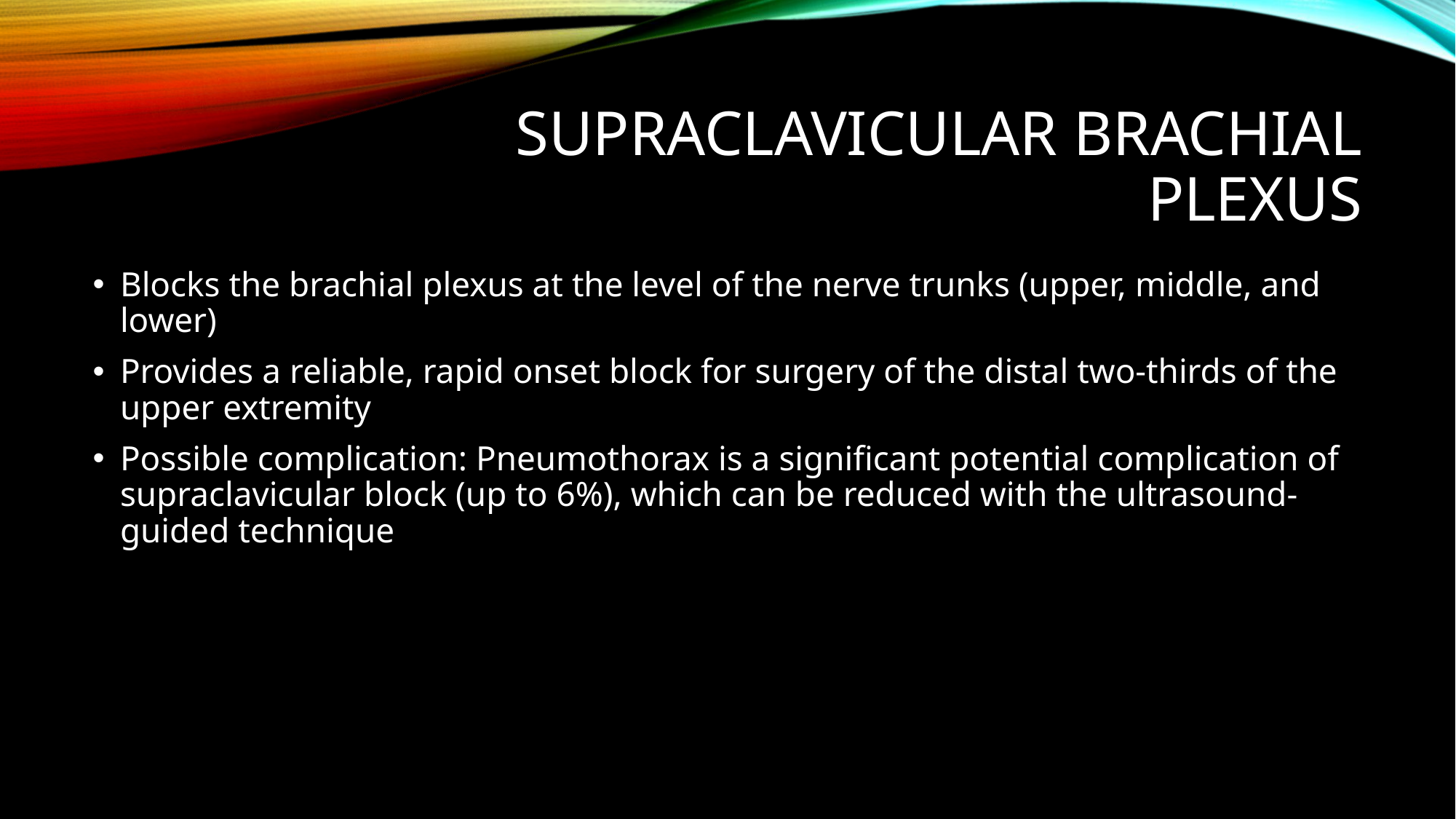

# Supraclavicular Brachial Plexus
Blocks the brachial plexus at the level of the nerve trunks (upper, middle, and lower)
Provides a reliable, rapid onset block for surgery of the distal two-thirds of the upper extremity
Possible complication: Pneumothorax is a significant potential complication of supraclavicular block (up to 6%), which can be reduced with the ultrasound-guided technique

## Slide 8
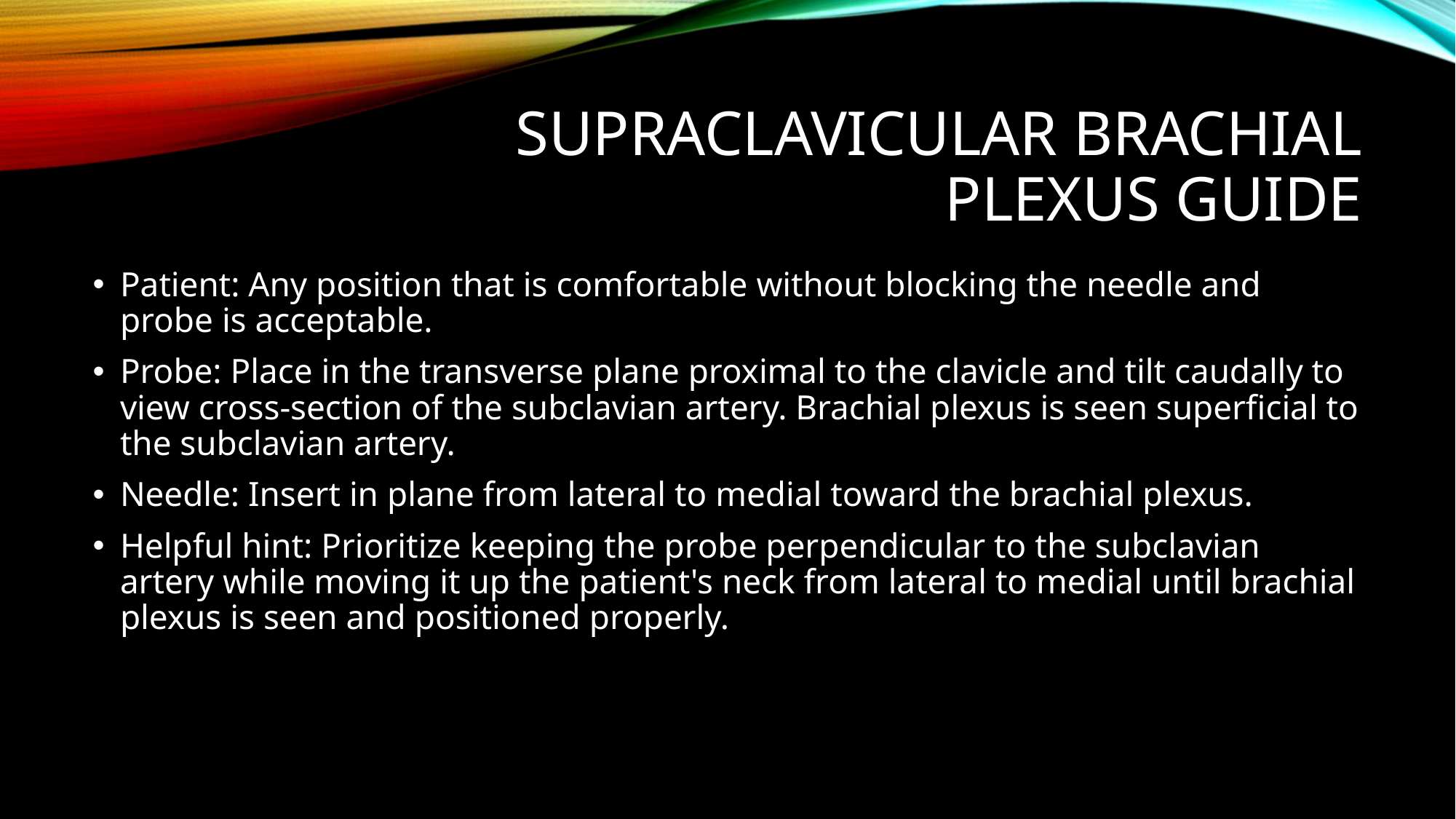

# Supraclavicular Brachial Plexus Guide
Patient: Any position that is comfortable without blocking the needle and probe is acceptable.
Probe: Place in the transverse plane proximal to the clavicle and tilt caudally to view cross-section of the subclavian artery. Brachial plexus is seen superficial to the subclavian artery.
Needle: Insert in plane from lateral to medial toward the brachial plexus.
Helpful hint: Prioritize keeping the probe perpendicular to the subclavian artery while moving it up the patient's neck from lateral to medial until brachial plexus is seen and positioned properly.

## Slide 9
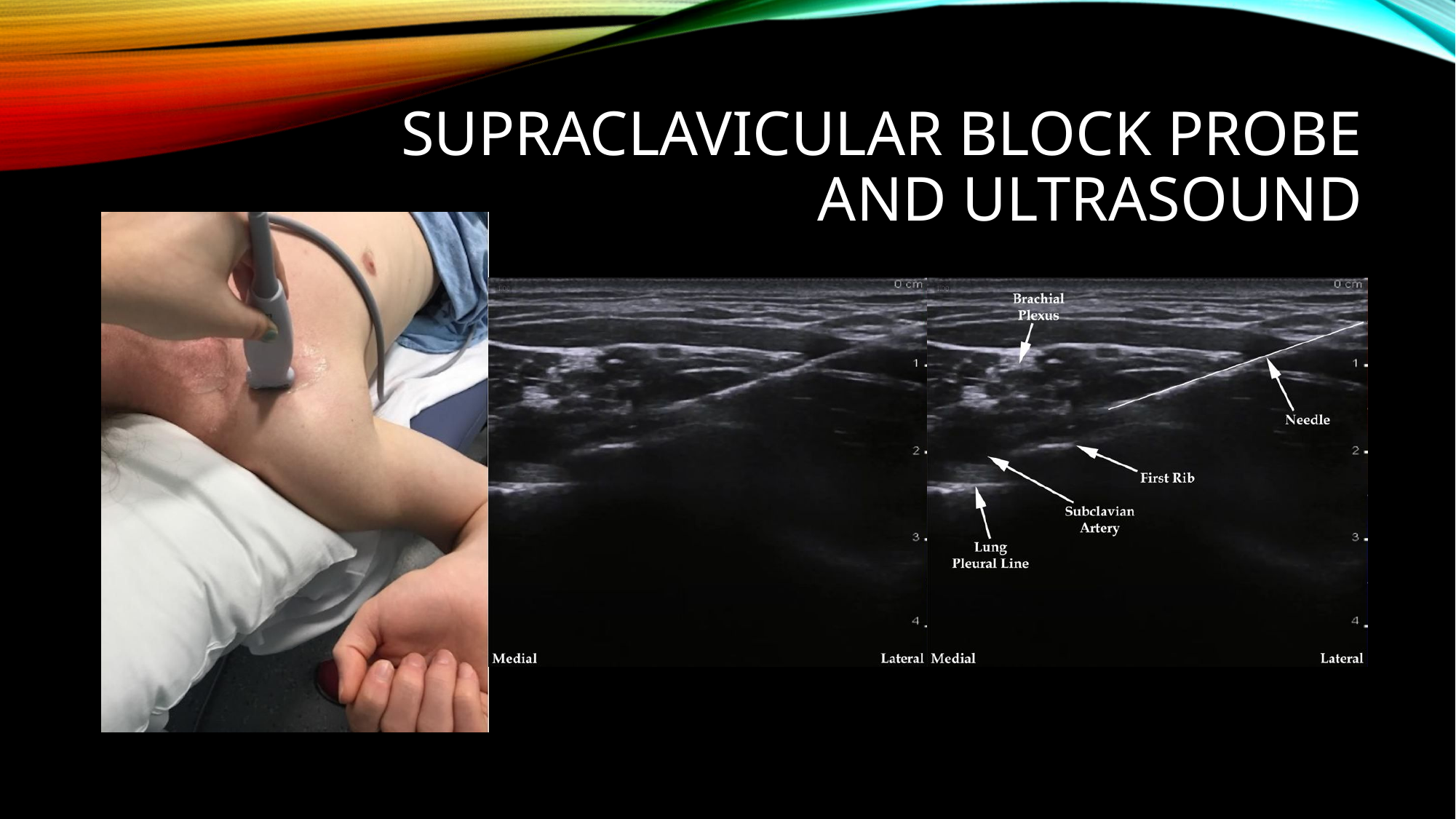

# Supraclavicular block Probe and ultrasound

## Slide 10
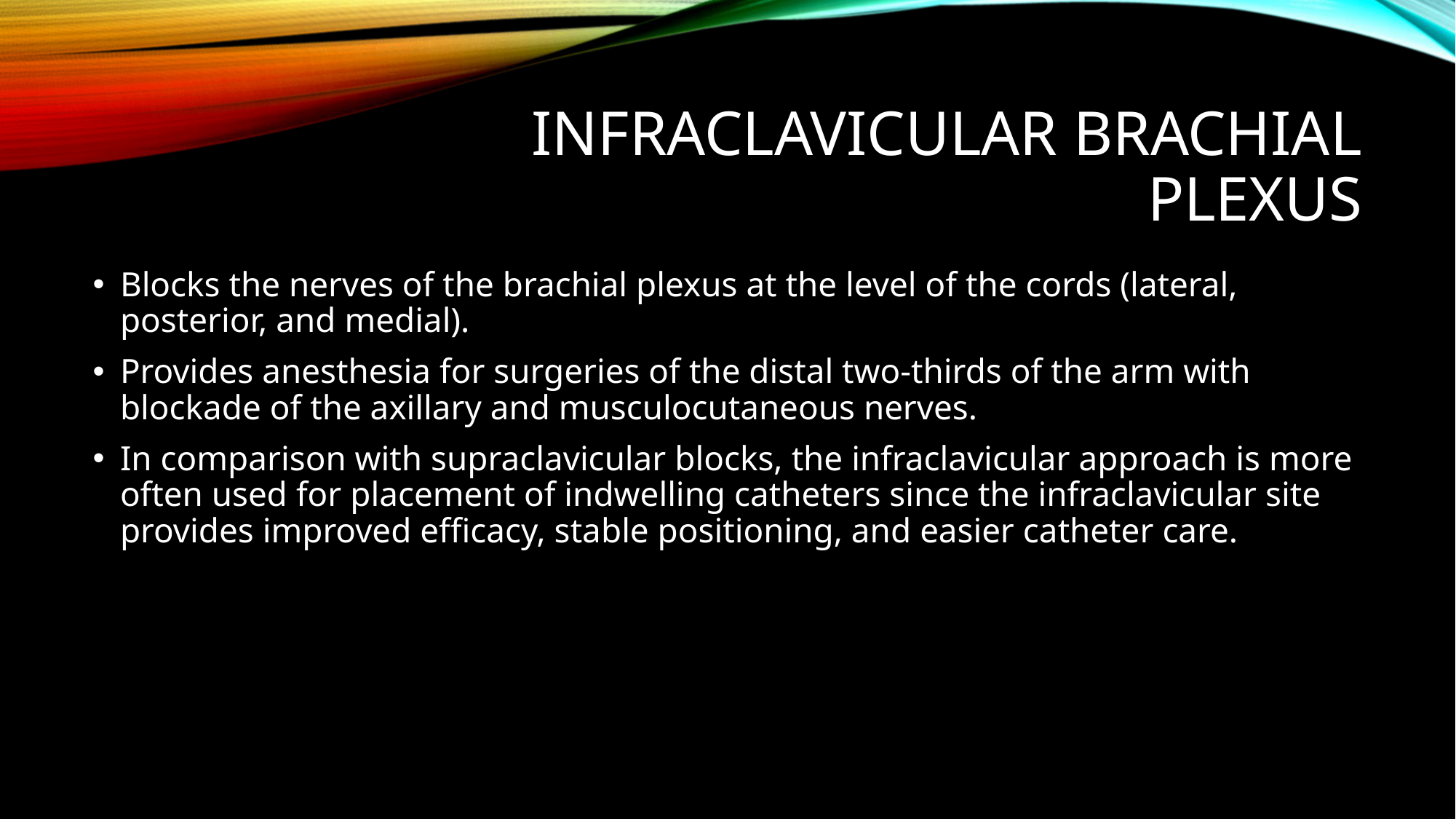

# Infraclavicular Brachial Plexus
Blocks the nerves of the brachial plexus at the level of the cords (lateral, posterior, and medial).
Provides anesthesia for surgeries of the distal two-thirds of the arm with blockade of the axillary and musculocutaneous nerves.
In comparison with supraclavicular blocks, the infraclavicular approach is more often used for placement of indwelling catheters since the infraclavicular site provides improved efficacy, stable positioning, and easier catheter care.

## Slide 11
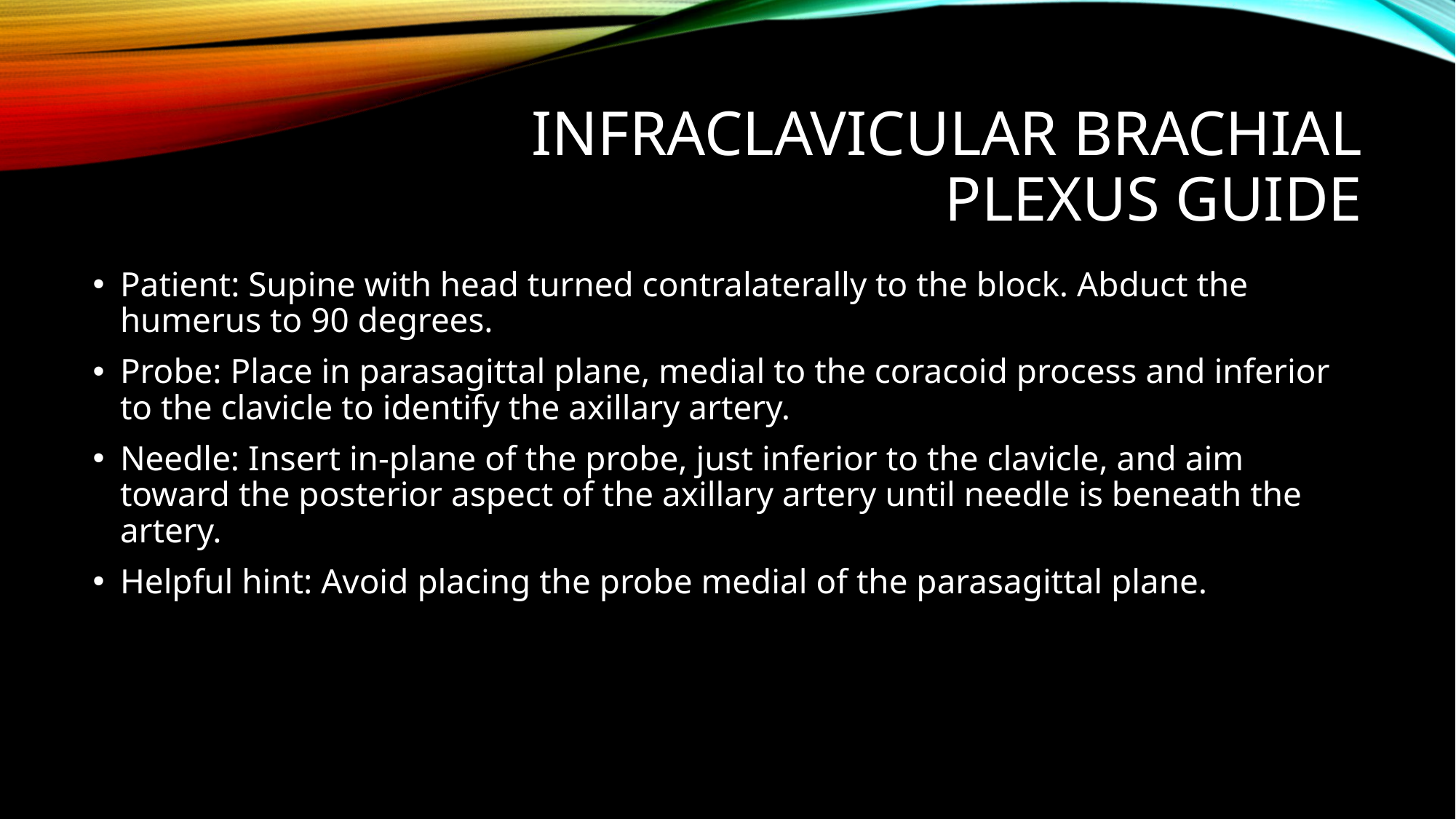

# Infraclavicular Brachial Plexus Guide
Patient: Supine with head turned contralaterally to the block. Abduct the humerus to 90 degrees.
Probe: Place in parasagittal plane, medial to the coracoid process and inferior to the clavicle to identify the axillary artery.
Needle: Insert in-plane of the probe, just inferior to the clavicle, and aim toward the posterior aspect of the axillary artery until needle is beneath the artery.
Helpful hint: Avoid placing the probe medial of the parasagittal plane.

## Slide 12
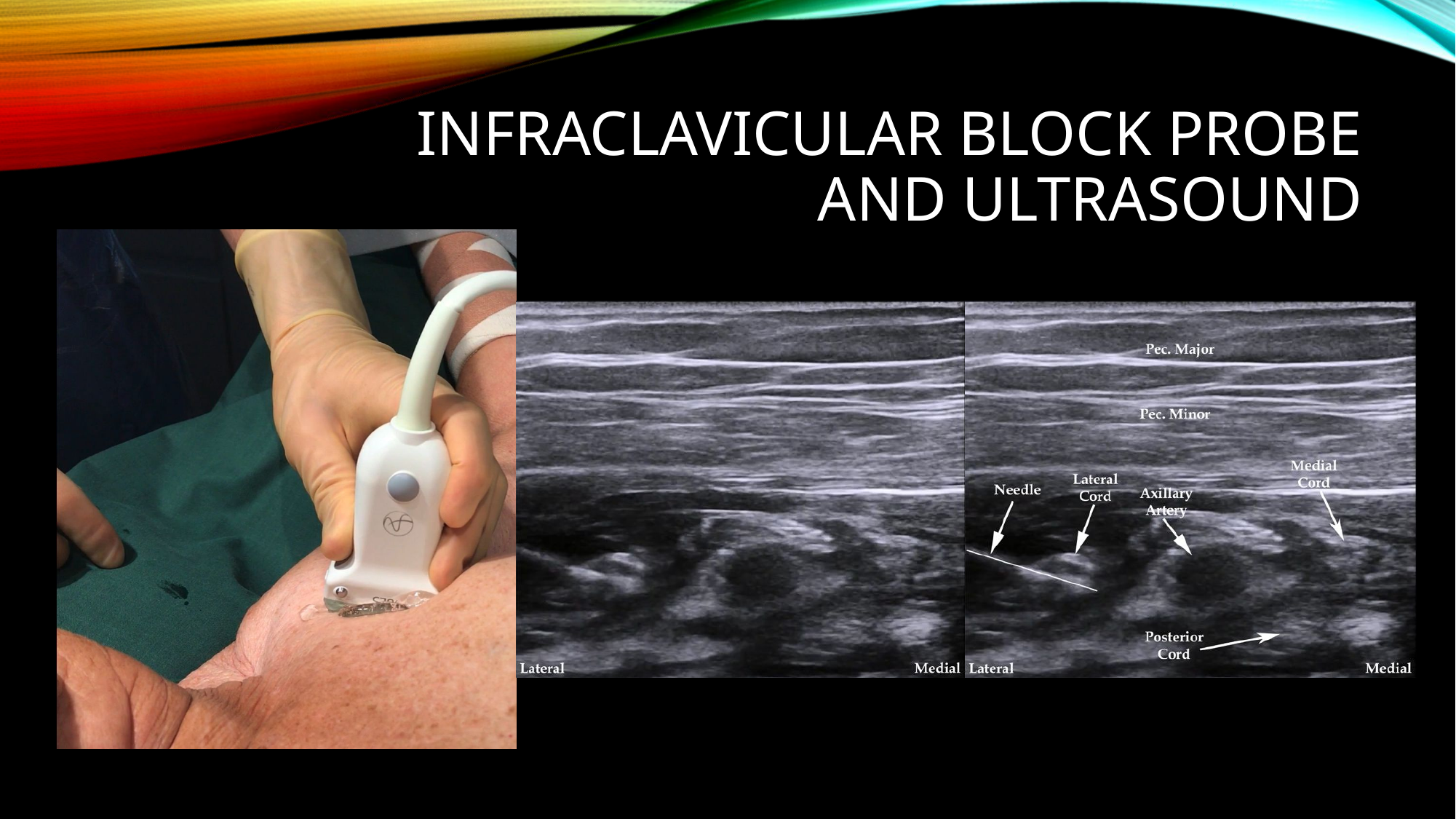

# infraclavicular block Probe and Ultrasound

## Slide 13
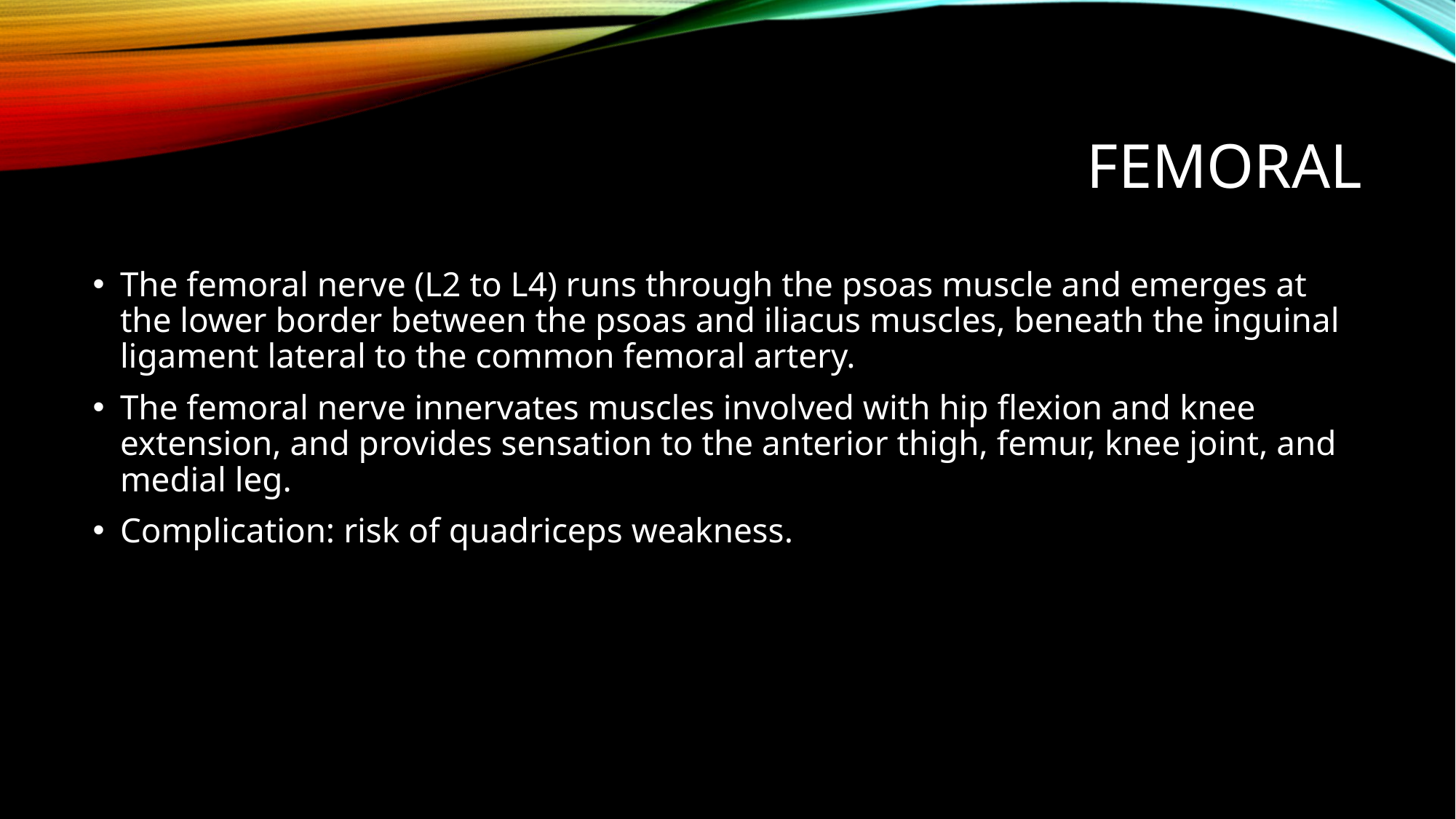

# Femoral
The femoral nerve (L2 to L4) runs through the psoas muscle and emerges at the lower border between the psoas and iliacus muscles, beneath the inguinal ligament lateral to the common femoral artery.
The femoral nerve innervates muscles involved with hip flexion and knee extension, and provides sensation to the anterior thigh, femur, knee joint, and medial leg.
Complication: risk of quadriceps weakness.

## Slide 14
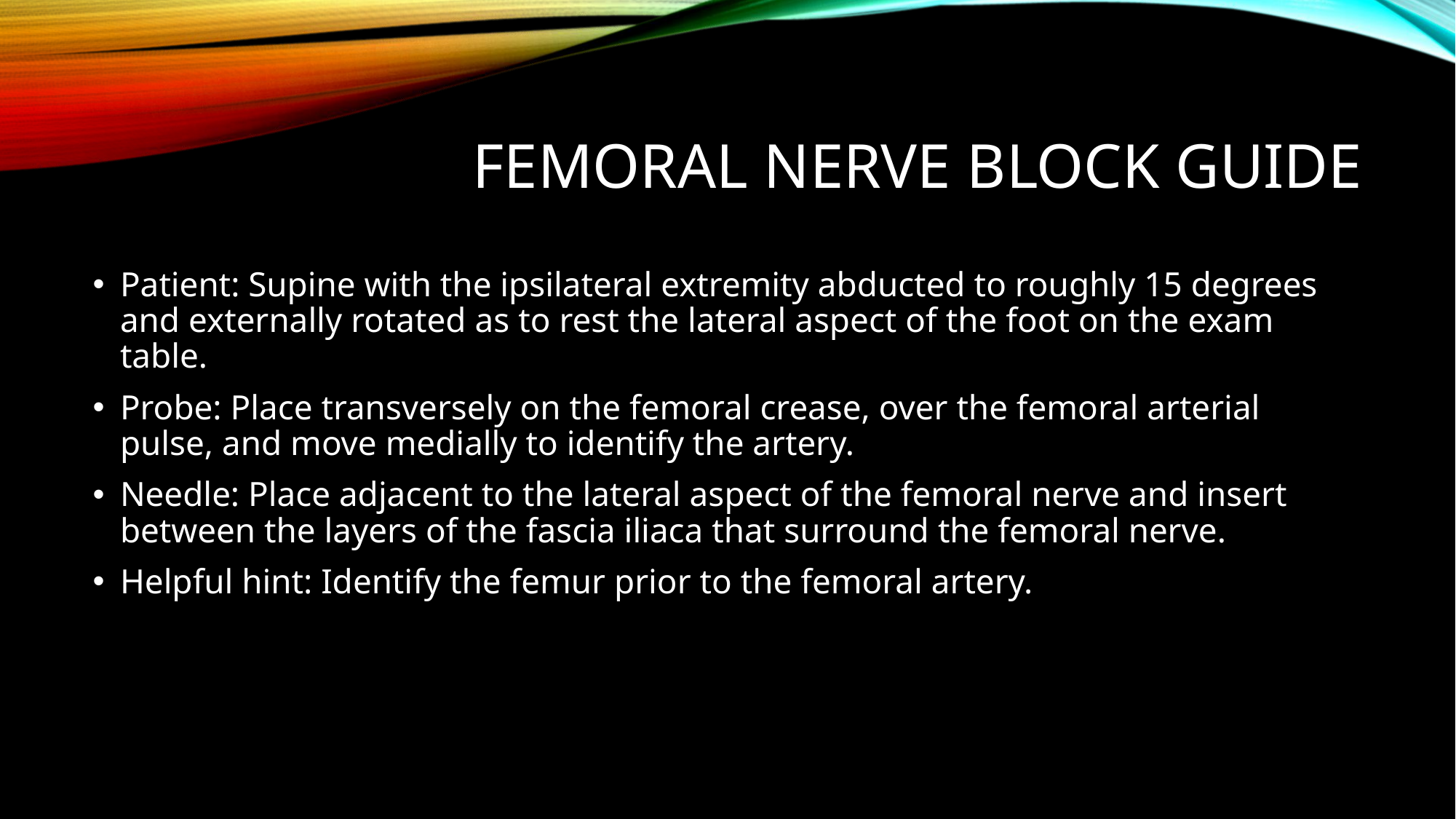

# Femoral Nerve Block Guide
Patient: Supine with the ipsilateral extremity abducted to roughly 15 degrees and externally rotated as to rest the lateral aspect of the foot on the exam table.
Probe: Place transversely on the femoral crease, over the femoral arterial pulse, and move medially to identify the artery.
Needle: Place adjacent to the lateral aspect of the femoral nerve and insert between the layers of the fascia iliaca that surround the femoral nerve.
Helpful hint: Identify the femur prior to the femoral artery.

## Slide 15
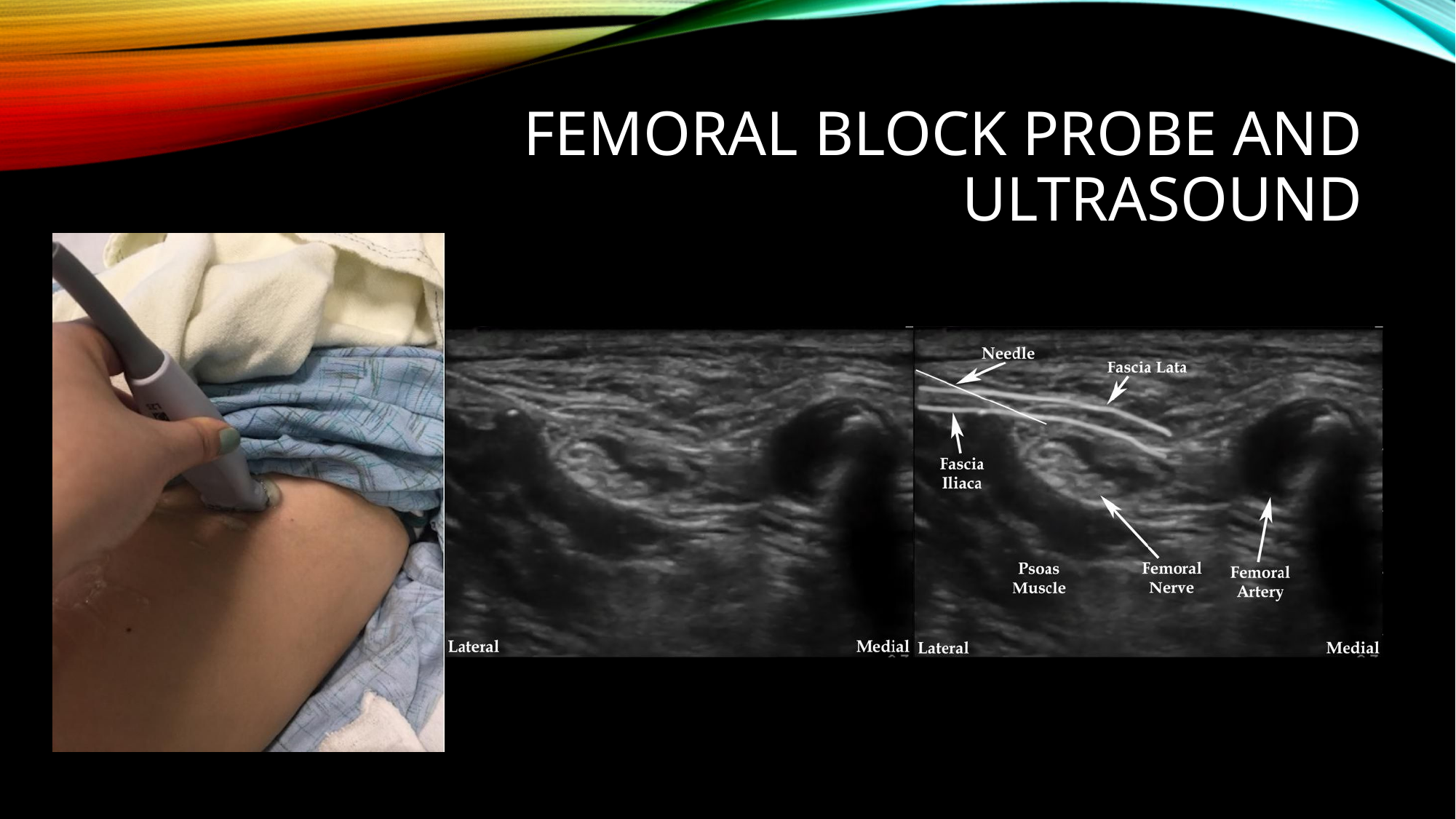

# Femoral block Probe and ultrasound

## Slide 16
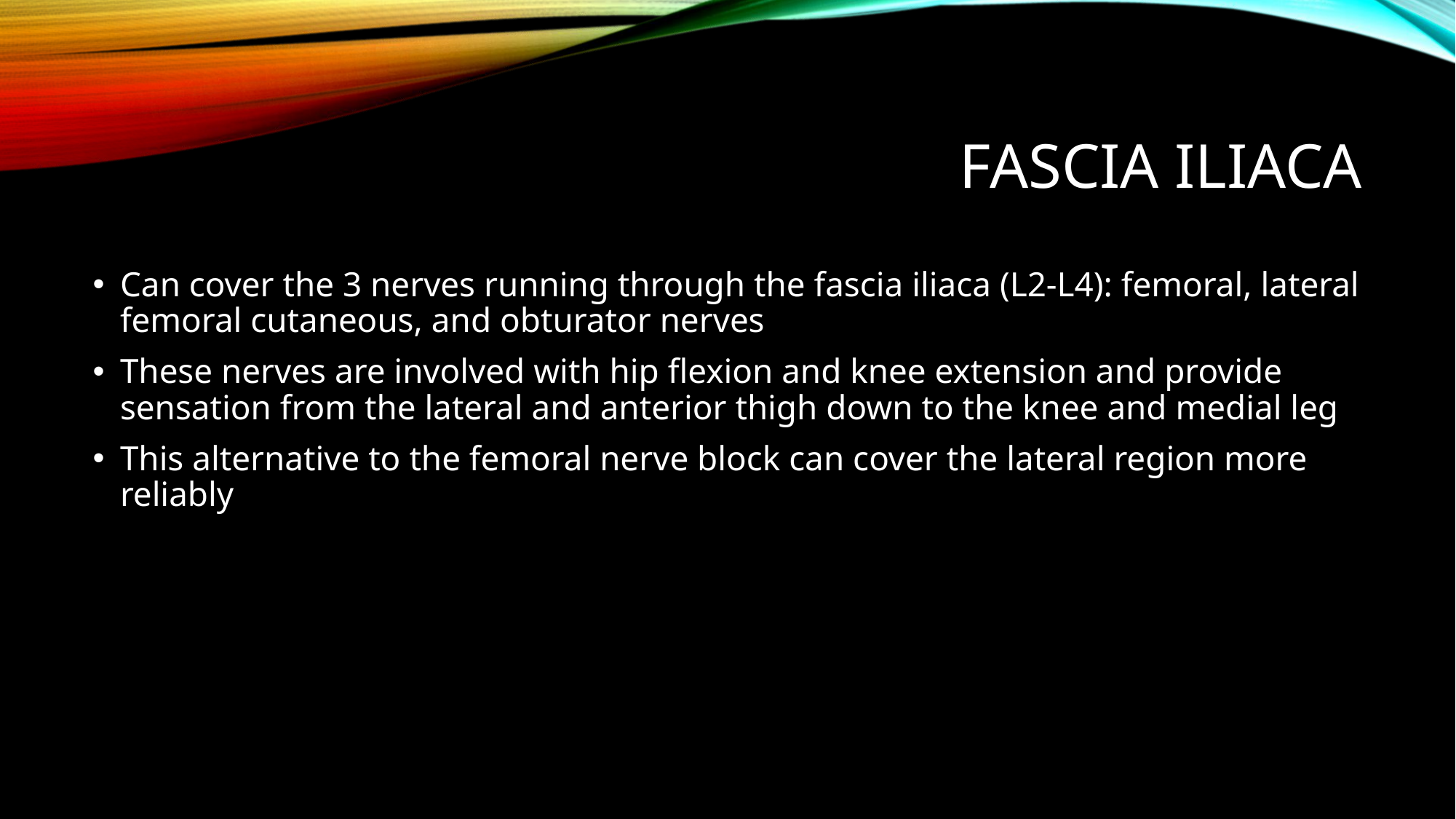

# Fascia Iliaca
Can cover the 3 nerves running through the fascia iliaca (L2-L4): femoral, lateral femoral cutaneous, and obturator nerves
These nerves are involved with hip flexion and knee extension and provide sensation from the lateral and anterior thigh down to the knee and medial leg
This alternative to the femoral nerve block can cover the lateral region more reliably

## Slide 17
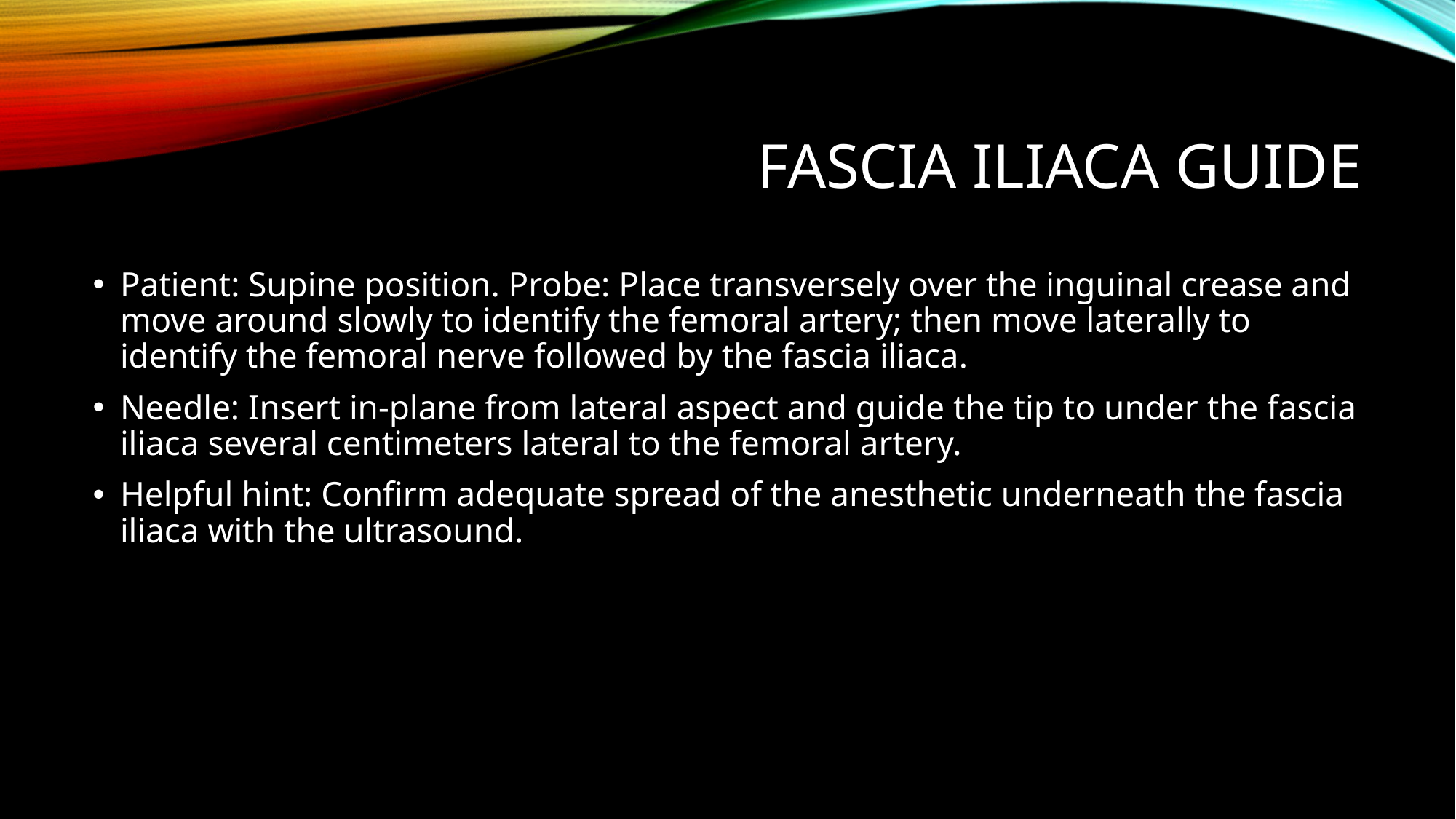

# Fascia Iliaca Guide
Patient: Supine position. Probe: Place transversely over the inguinal crease and move around slowly to identify the femoral artery; then move laterally to identify the femoral nerve followed by the fascia iliaca.
Needle: Insert in-plane from lateral aspect and guide the tip to under the fascia iliaca several centimeters lateral to the femoral artery.
Helpful hint: Confirm adequate spread of the anesthetic underneath the fascia iliaca with the ultrasound.

## Slide 18
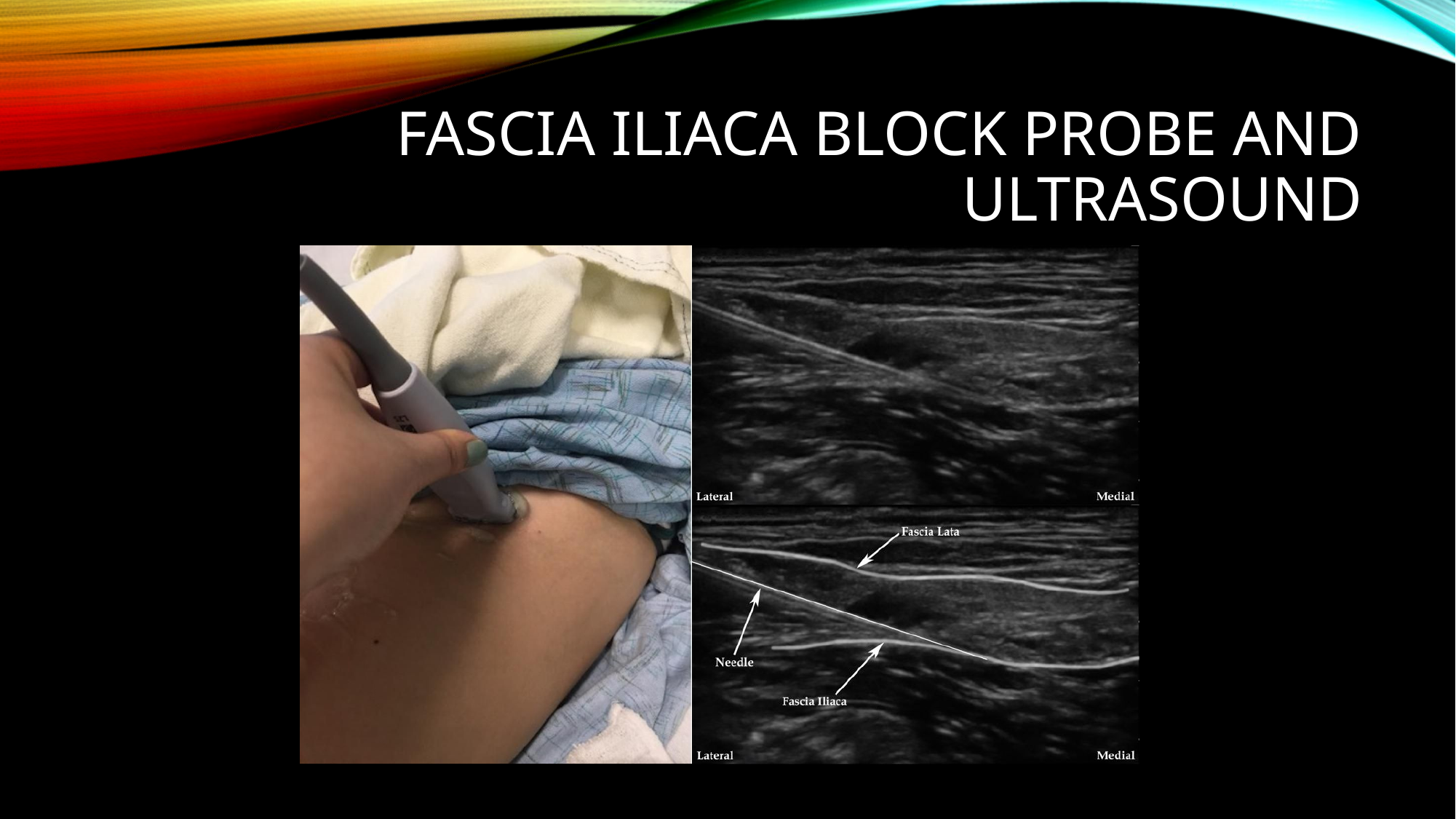

# Fascia iliaca block Probe and ultrasound

## Slide 19
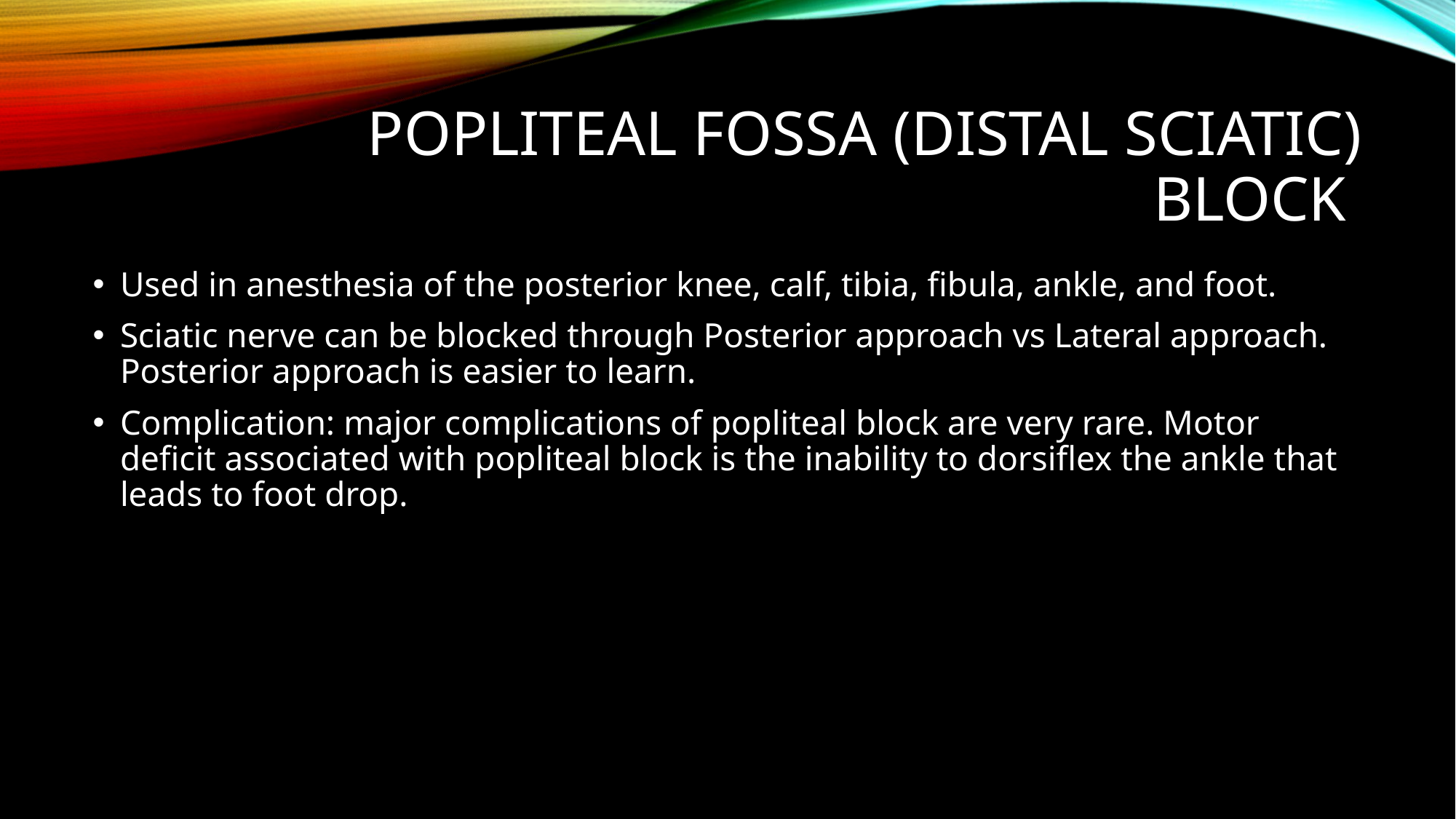

# Popliteal Fossa (Distal Sciatic) Block
Used in anesthesia of the posterior knee, calf, tibia, fibula, ankle, and foot.
Sciatic nerve can be blocked through Posterior approach vs Lateral approach. Posterior approach is easier to learn.
Complication: major complications of popliteal block are very rare. Motor deficit associated with popliteal block is the inability to dorsiflex the ankle that leads to foot drop.

## Slide 20
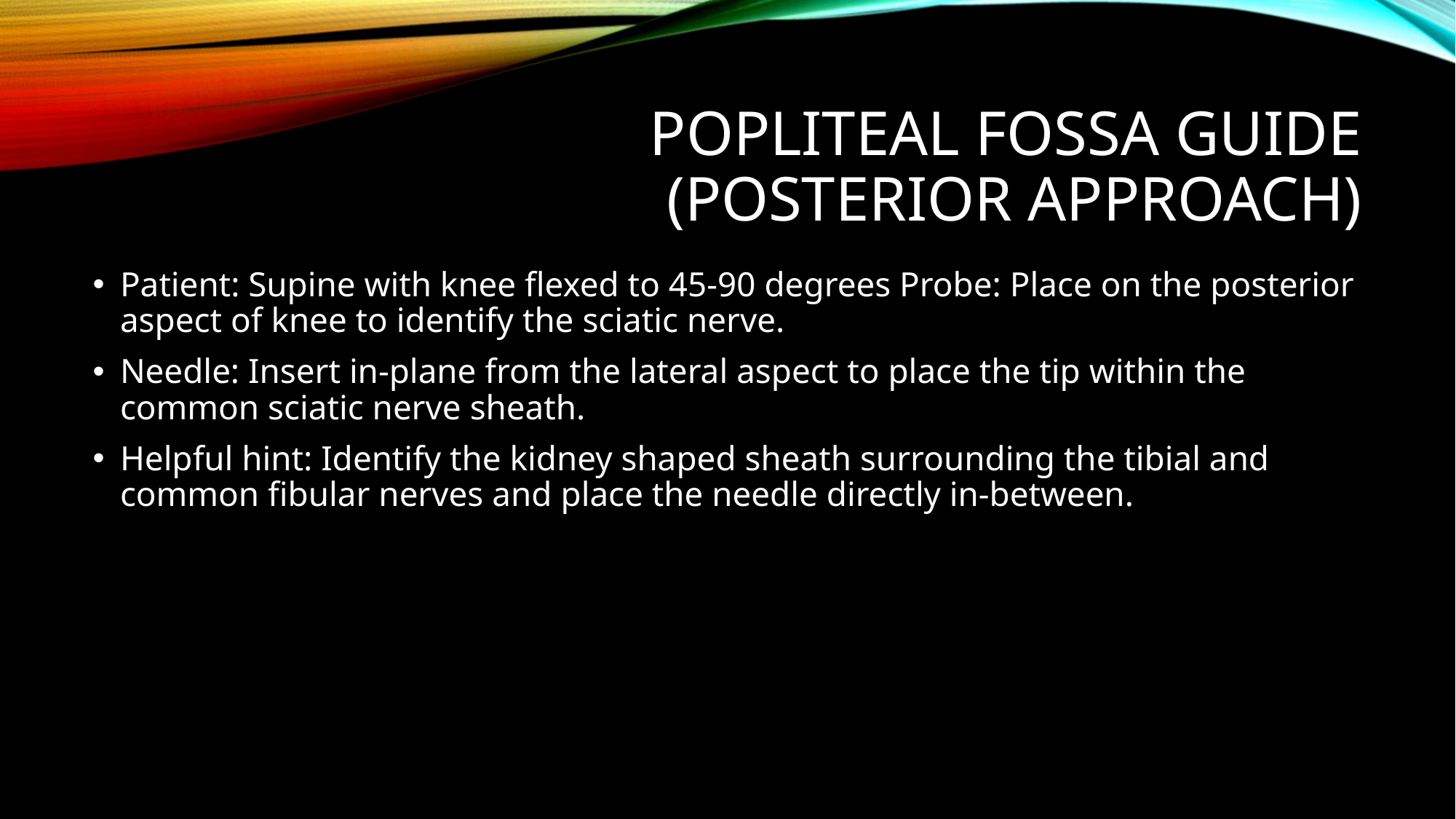

# Popliteal Fossa Guide (Posterior Approach)
Patient: Supine with knee flexed to 45-90 degrees Probe: Place on the posterior aspect of knee to identify the sciatic nerve.
Needle: Insert in-plane from the lateral aspect to place the tip within the common sciatic nerve sheath.
Helpful hint: Identify the kidney shaped sheath surrounding the tibial and common fibular nerves and place the needle directly in-between.

## Slide 21
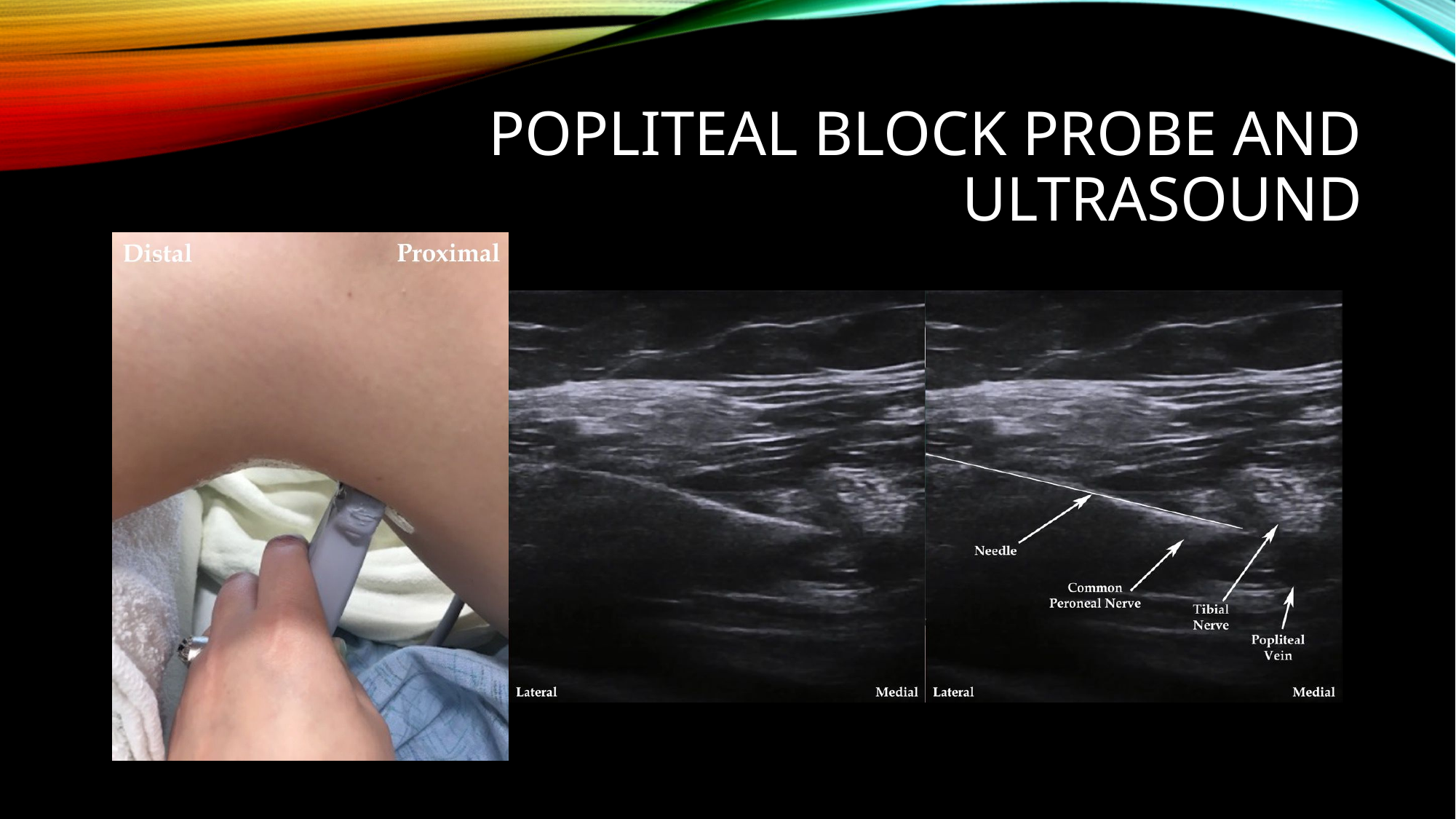

# Popliteal Block Probe and ultrasound

## Slide 22
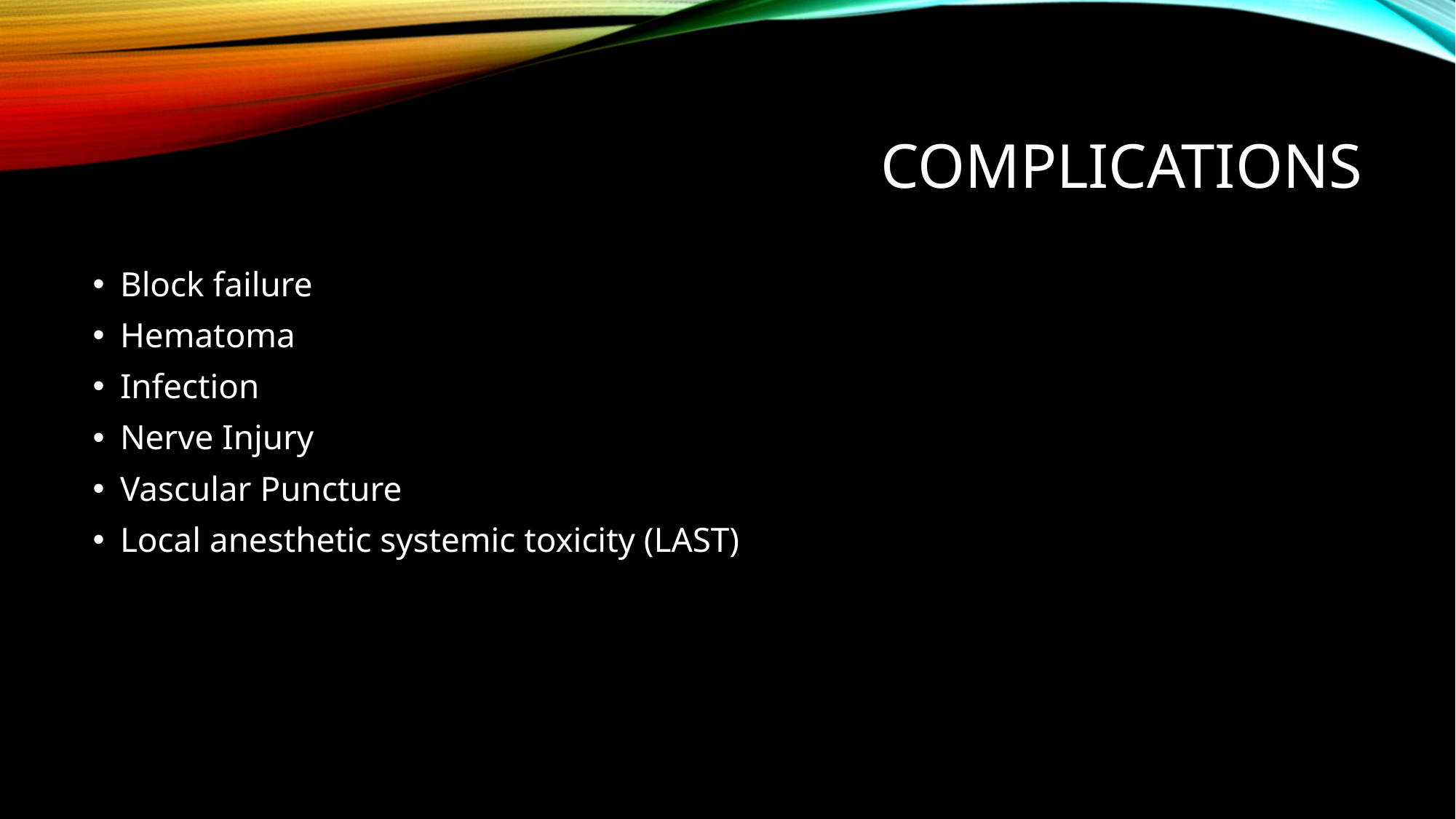

# Complications
Block failure
Hematoma
Infection
Nerve Injury
Vascular Puncture
Local anesthetic systemic toxicity (LAST)

## Slide 23
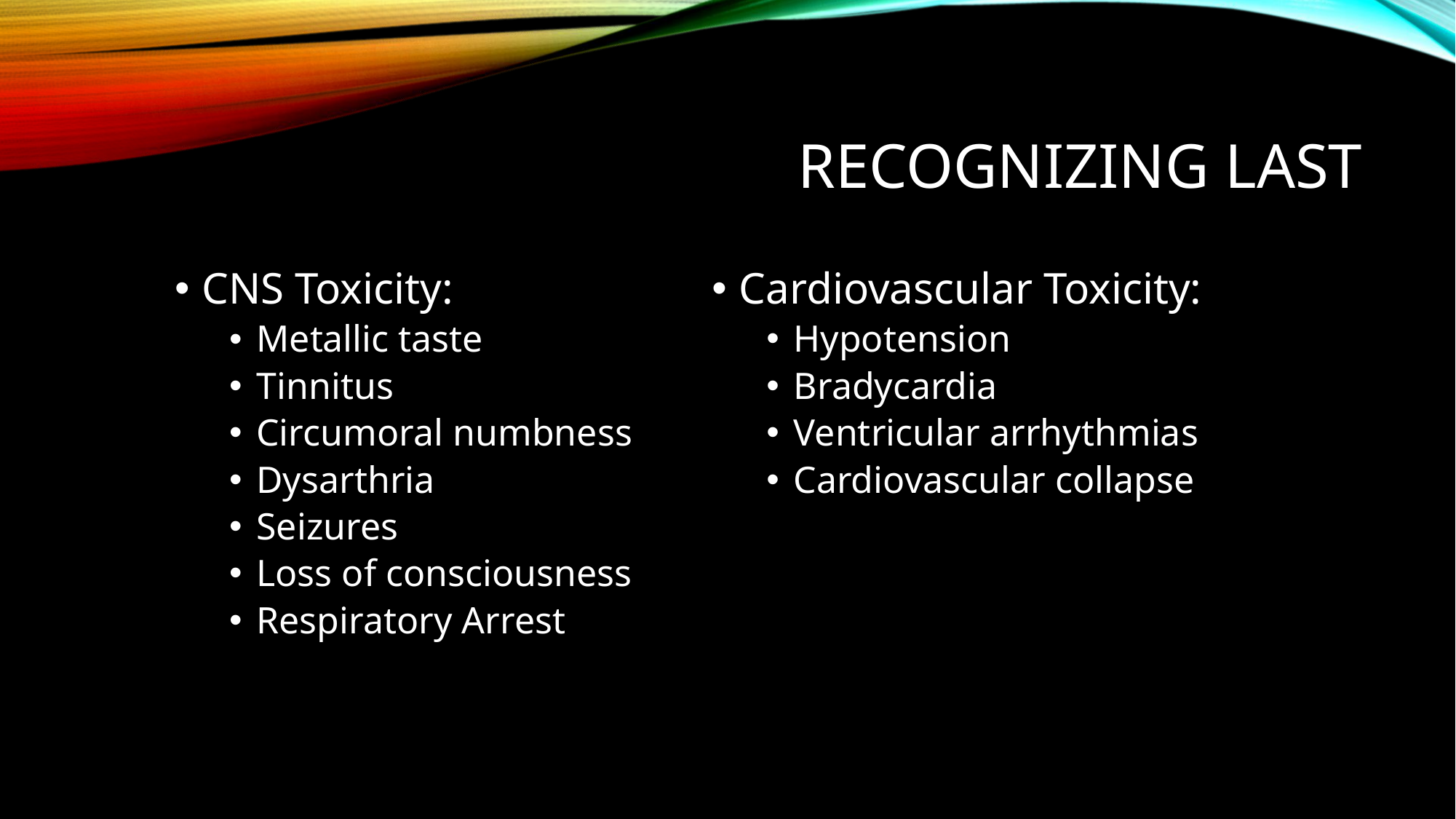

# Recognizing LAST
CNS Toxicity:
Metallic taste
Tinnitus
Circumoral numbness
Dysarthria
Seizures
Loss of consciousness
Respiratory Arrest
Cardiovascular Toxicity:
Hypotension
Bradycardia
Ventricular arrhythmias
Cardiovascular collapse

## Slide 24
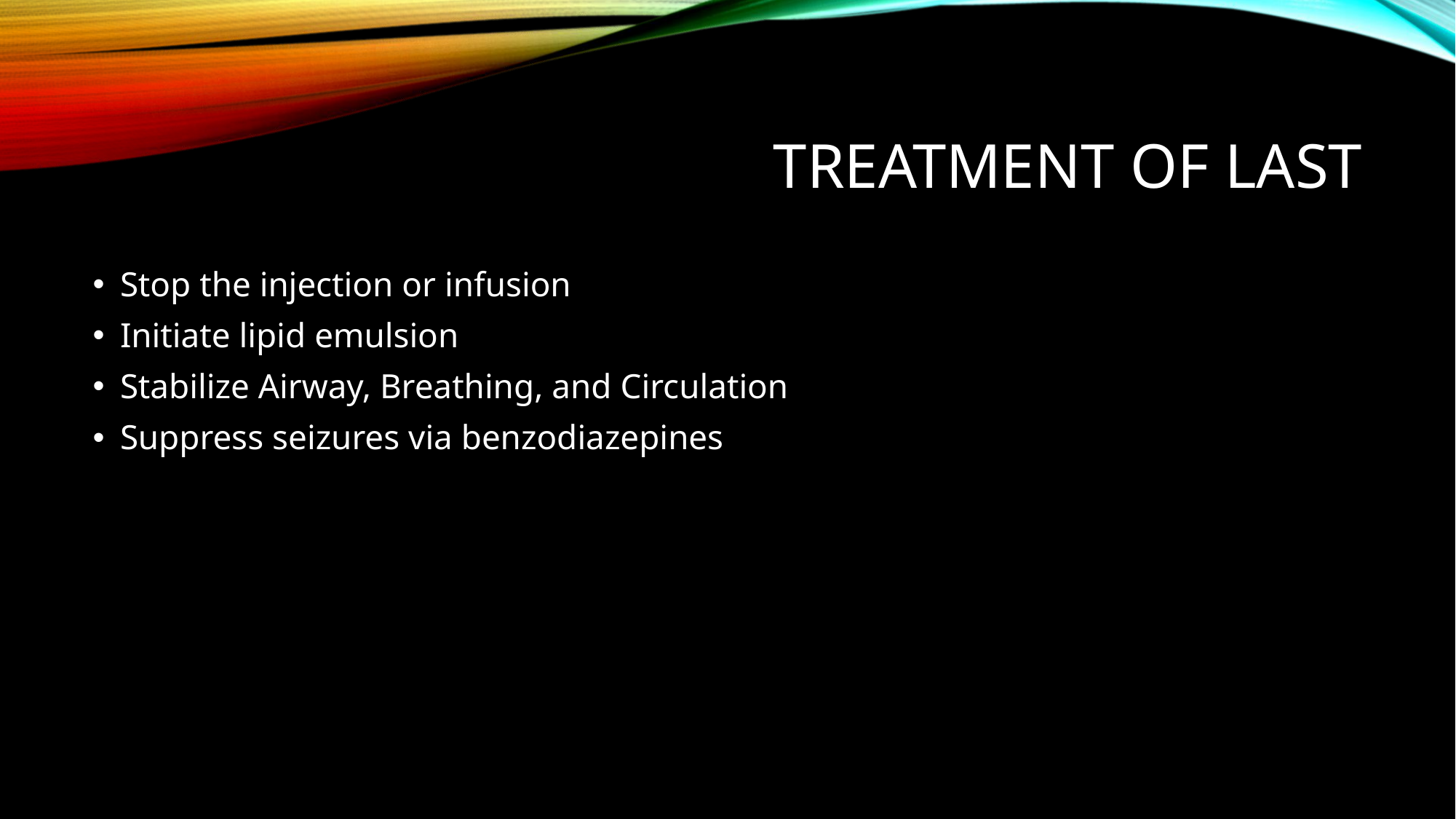

# Treatment of LAST
Stop the injection or infusion
Initiate lipid emulsion
Stabilize Airway, Breathing, and Circulation
Suppress seizures via benzodiazepines

## Slide 25
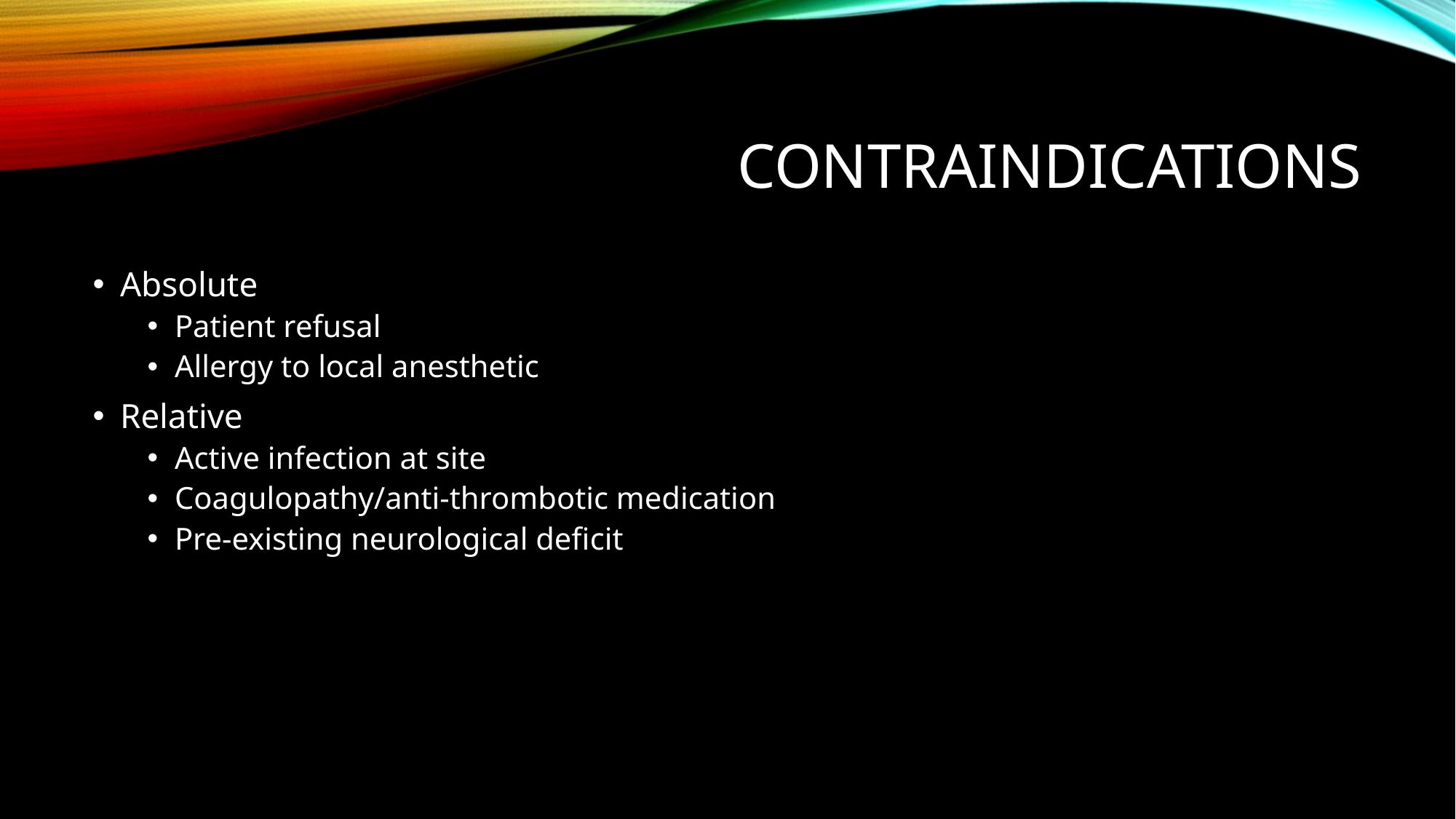

# Contraindications
Absolute
Patient refusal
Allergy to local anesthetic
Relative
Active infection at site
Coagulopathy/anti-thrombotic medication
Pre-existing neurological deficit

## Slide 26
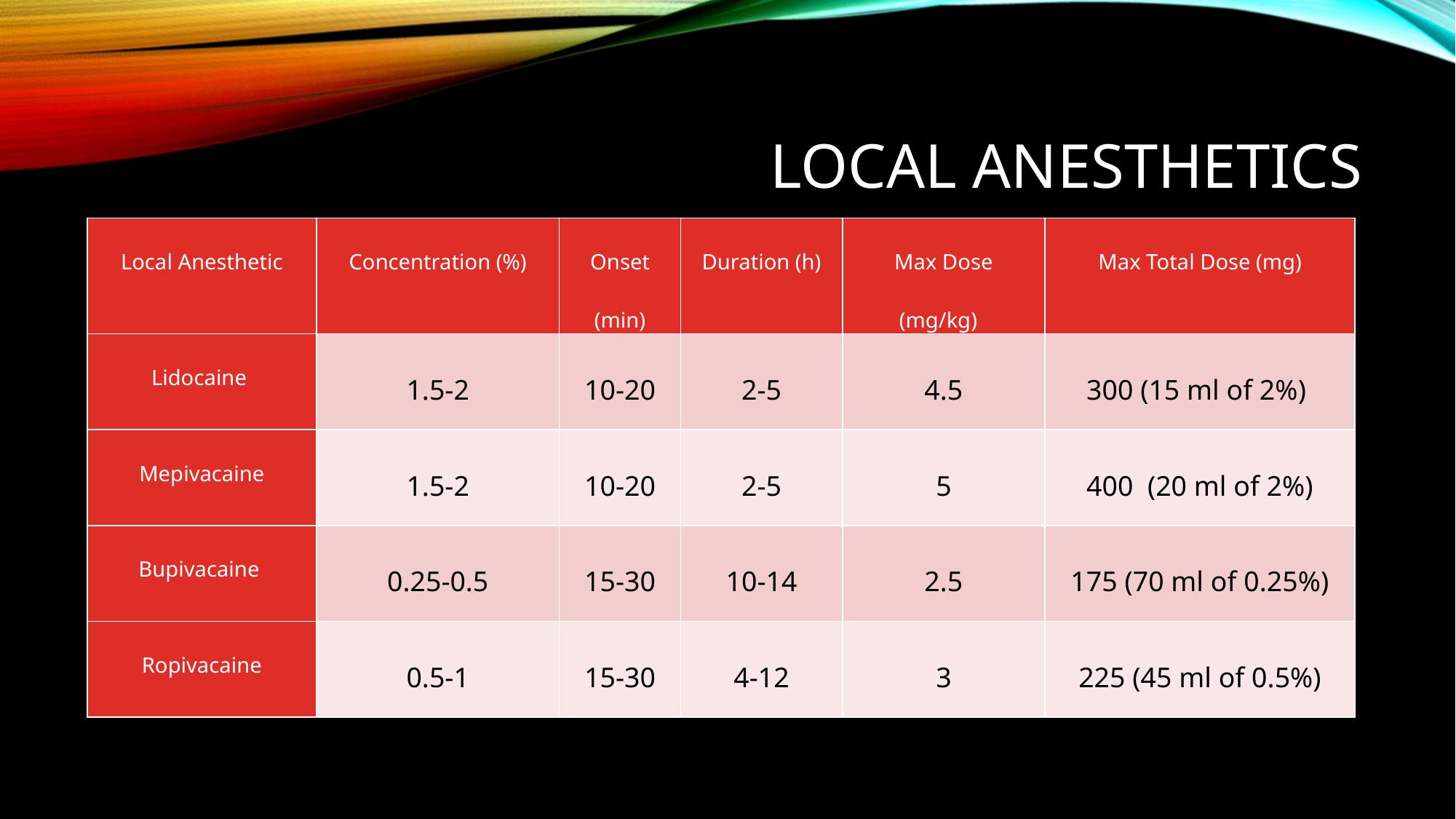

# Local Anesthetics
| Local Anesthetic | Concentration (%) | Onset (min) | Duration (h) | Max Dose (mg/kg) | Max Total Dose (mg) |
| --- | --- | --- | --- | --- | --- |
| Lidocaine | 1.5-2 | 10-20 | 2-5 | 4.5 | 300 (15 ml of 2%) |
| Mepivacaine | 1.5-2 | 10-20 | 2-5 | 5 | 400 (20 ml of 2%) |
| Bupivacaine | 0.25-0.5 | 15-30 | 10-14 | 2.5 | 175 (70 ml of 0.25%) |
| Ropivacaine | 0.5-1 | 15-30 | 4-12 | 3 | 225 (45 ml of 0.5%) |

## Slide 27
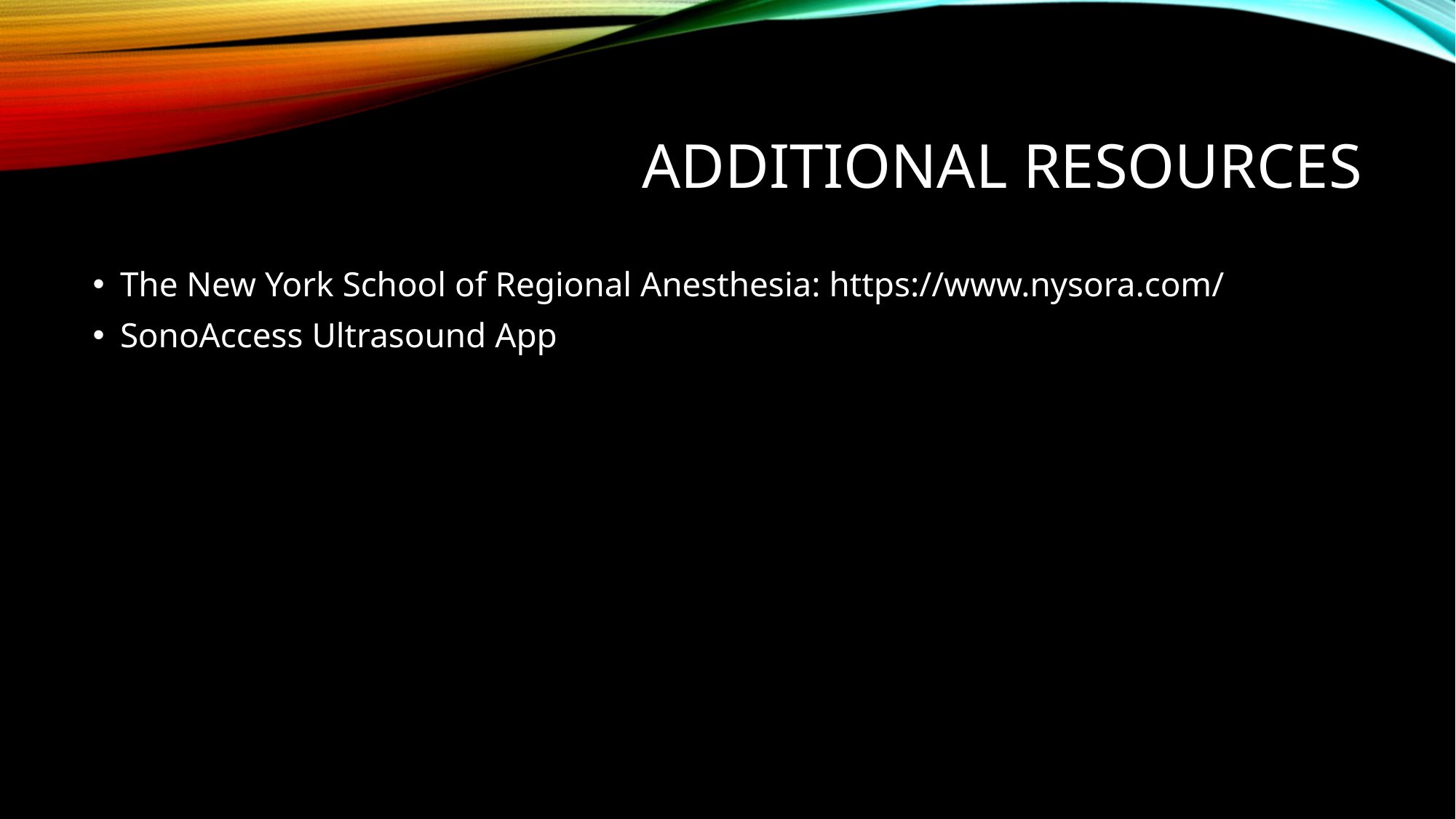

# Additional Resources
The New York School of Regional Anesthesia: https://www.nysora.com/
SonoAccess Ultrasound App

## Slide 28
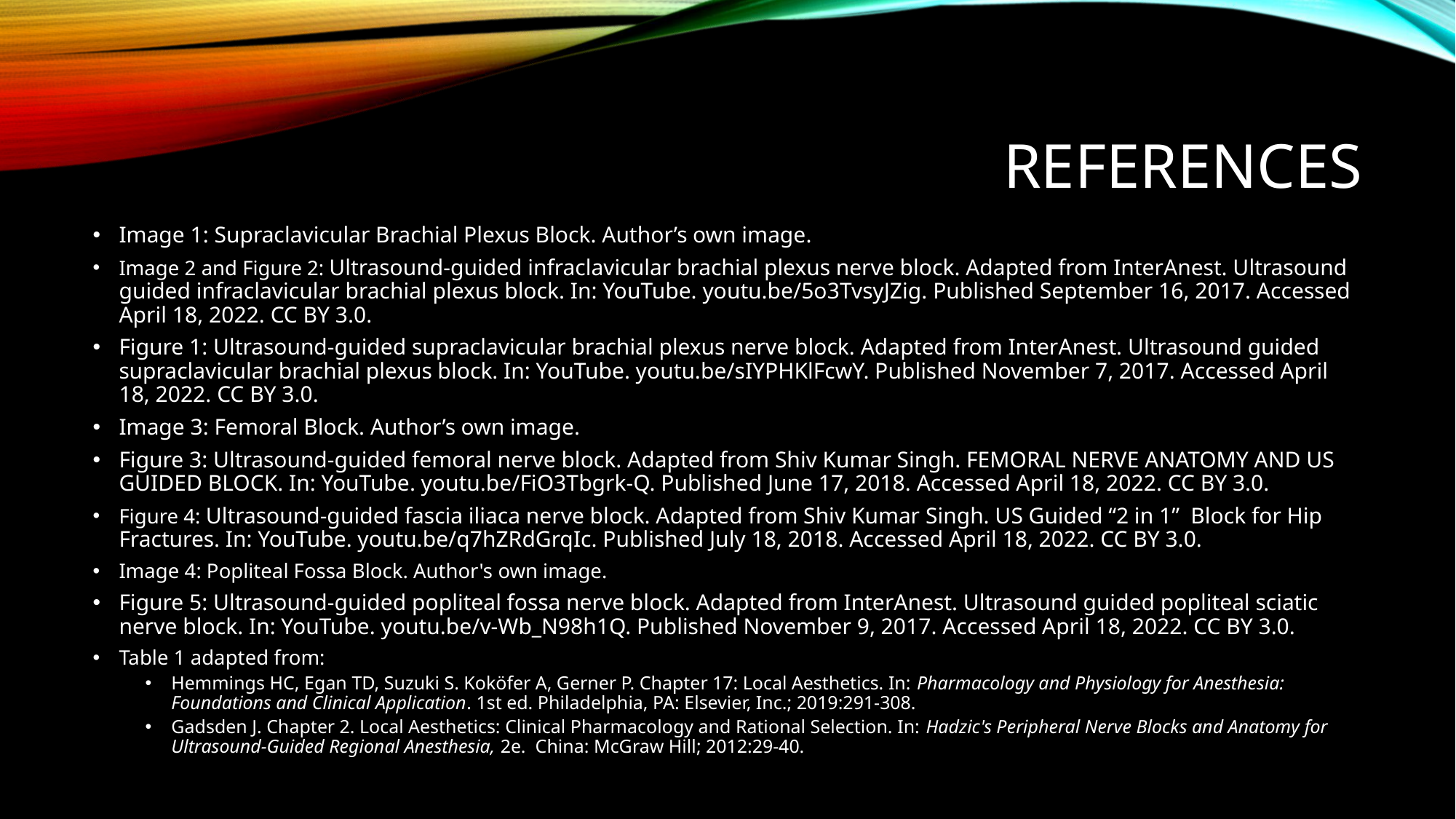

# References
Image 1: Supraclavicular Brachial Plexus Block. Author’s own image.
Image 2 and Figure 2: Ultrasound-guided infraclavicular brachial plexus nerve block. Adapted from InterAnest. Ultrasound guided infraclavicular brachial plexus block. In: YouTube. youtu.be/5o3TvsyJZig. Published September 16, 2017. Accessed April 18, 2022. CC BY 3.0.
Figure 1: Ultrasound-guided supraclavicular brachial plexus nerve block. Adapted from InterAnest. Ultrasound guided supraclavicular brachial plexus block. In: YouTube. youtu.be/sIYPHKlFcwY. Published November 7, 2017. Accessed April 18, 2022. CC BY 3.0.
Image 3: Femoral Block. Author’s own image.
Figure 3: Ultrasound-guided femoral nerve block. Adapted from Shiv Kumar Singh. FEMORAL NERVE ANATOMY AND US GUIDED BLOCK. In: YouTube. youtu.be/FiO3Tbgrk-Q. Published June 17, 2018. Accessed April 18, 2022. CC BY 3.0.
Figure 4: Ultrasound-guided fascia iliaca nerve block. Adapted from Shiv Kumar Singh. US Guided “2 in 1” Block for Hip Fractures. In: YouTube. youtu.be/q7hZRdGrqIc. Published July 18, 2018. Accessed April 18, 2022. CC BY 3.0.
Image 4: Popliteal Fossa Block. Author's own image.
Figure 5: Ultrasound-guided popliteal fossa nerve block. Adapted from InterAnest. Ultrasound guided popliteal sciatic nerve block. In: YouTube. youtu.be/v-Wb_N98h1Q. Published November 9, 2017. Accessed April 18, 2022. CC BY 3.0.
Table 1 adapted from:
Hemmings HC, Egan TD, Suzuki S. Koköfer A, Gerner P. Chapter 17: Local Aesthetics. In: Pharmacology and Physiology for Anesthesia: Foundations and Clinical Application. 1st ed. Philadelphia, PA: Elsevier, Inc.; 2019:291-308.
Gadsden J. Chapter 2. Local Aesthetics: Clinical Pharmacology and Rational Selection. In: Hadzic's Peripheral Nerve Blocks and Anatomy for Ultrasound-Guided Regional Anesthesia, 2e. China: McGraw Hill; 2012:29-40.
